# Phytochemical analysis and biological study on *Sinapis alba* L. seeds extract incorporated with metal nanoparticles, in vitro approach

**DOI:** 10.1038/s41598-025-95347-6

**Published:** 2025-04-21

**Authors:** Wael Mahmoud Aboulthana, Amal M. El-Feky, Noha El-Sayed Ibrahim, Ahmed A. F. Soliman, Ahmed Mahmoud Youssef

**Affiliations:** 1 Biochemistry Department, Biotechnology Research Institute, National Research Centre, 33 El Bohouth St. (Former El Tahrir St.), P.O. 12622, Dokki, Giza Egypt; 2 Pharmacognosy Department, Pharmaceutical and Drug Industries Research Institute, National Research Centre, 33 El Bohouth St. (Former El Tahrir St.), P.O. 12622, Dokki, Giza Egypt; 3 Microbial Biotechnology Department, Biotechnology Research Institute, National Research Centre, 33 El Bohouth St. (Former El Tahrir St.), P.O. 12622, Dokki, Giza Egypt; 4 Packaging Materials Department, National Research Centre, 33 El Bohouth St. (Former El Tahrir St.), P.O. 12622, Dokki, Giza Egypt

**Keywords:** *Sinapis alba* L., Seeds, Green Nanotechnology, Metal Nanoparticles, Anti-diabetic Activity, Cytotoxic Activity, Biochemistry, Biological techniques, Biotechnology

## Abstract

White mustard (*Sinapis alba* L.) seeds are the most commonly used mustard species in herbal medicine to treat a wide range of inflammatory disorders. Due to its increased bioavailability and lower toxicity, the green biosynthesis of metal nanoparticles (M-NPs) utilizing plant extract as a capping agent has been demonstrated over a number of years. Thus, the current study sought to examine the *in vitro* biological activity of copper oxide nanoparticles  (CuO-NPs) and selenium nanoparticles (Se-NPs) that were biosynthesized using aqueous, methanolic, and petroleum ether extracts from *S. alba* seeds. Phytochemical and *in vitro *biological activities (antioxidant, scavenging, anti-diabetic, anti-acetylcholinesterase, anti-arthritic, anti-inflammatory, and cytotoxic activities) were assayed in all prepared extracts before and after being used for the biosynthesis of the M-NPs. It was found that the total methanolic extract possessed the highest biological activities compared to other native extracts. The LC-ESI-MS/MS analysis of secondary metabolites showed that the total methanolic extract contained 7 phenolic acids and 9 flavonoid aglycones. This helped find the active ingredients. We characterized 8 phenolic acid derivatives, 7 flavonoid glycosides, 4 aliphatic glucosinolates, and 3 aromatic aryl glucosinolates in the aqueous extract. Furthermore, the methanolic extract contains the highest concentrations of total polyphenols, condensed tannins, and total flavonoid compounds. The biosynthesized Se-NPs using methanolic extract showed higher *in vitro* biological activities compared to those of the biosynthesized CuO-NPs. The median lethal dose (LD_50_) showed that the biosynthesized Se-NPs using the studied extracts appeared safer compared to those of the biosynthesized CuO-NPs. The findings of this study concluded that the total methanolic extract is the most suitable bioresource for biosynthesizing Se-NPs through green nanotechnology, with higher biological efficiency in relation to its metabolite fingerprint.

## Introduction

Plant-derived herbal medicines are widely used as a therapeutic tool for treating various chronic diseases^1^. The edible medicinal plants, which are categorized as a safe choice for their antioxidant, anti-inflammatory, and anticancer properties, are used as adjuvants for cancer therapy^2^.

Mustard belongs to the family *Brassicaceae*, native to the Mediterranean region and grown widely around the world for thousands of years. It is categorized as a multifunctional plant, especially the seeds, which have a high fat content of 23–47%, dominated by erucic acid (26.5–36.5%), in addition to the high protein content (18–24%), which can serve as a source of food-grade vegetable protein^3^. Depending on the species, the proportion of other fatty acids varies. For example, white mustard oil has a high oleic acid content (22%) and linolenic acid is also found at significant levels (9–15%)^4^.

White mustard (*Sinapis alba* L.) seeds are the most commonly used mustard species in Europe. They are commonly used as a food flavoring and also as herbal medicine to treat arthritis, cough, sore throat, and diabetes^5^. They are characterized by the presence of special compounds namely glucosinolates, responsible for the flavor of mustard. Sinigrin is the main glucosinolate compound found in mustard, followed by sinalbin and glucobrassicin, which are degraded into isothiocyanates and allyl isothiocyanate by the enzymatic action of intestinal flora^6^. Isothiocyanates and allyl isothiocyanate exhibit their chemopreventive effects by inhibiting the metabolic activation of carcinogens by cytochrome P450s and inducing detoxifying and cellular defensive enzymes. They have also shown promising results in inhibiting the growth of foodborne pathogens, indicating their potential for use as antimicrobial and antifungal agents in various foods^7,8^.

Most bioactive compounds have a large molecular weight, which affects their absorption through cellular membranes, decreasing their bioavailability and efficiency^9^. Nanotechnology is used to solve such problems by increasing bioavailability, efficiency, stability, solubility, decreasing toxicities, and achieving targeted release at the appropriate action site^10,11^. Therefore, incorporating metal nanoparticles (M-NPs) into plant extracts may be considered one of the most promising solutions to overcome the problem of poor solubility, distribution, and absorption properties of organic compounds^12^.

This was supported by a number of recent studies that were concerned with incorporating silver nanoparticles (Ag-NPs) into *Croton tiglium* seeds^13^ and *Moringa oleifera* leaves extracts^14^ to be used against colon cancer in rats. Additionally, gold nanoparticles (Au-NPs) were incorporated into *Bauhinia variegata* leaves extracts and used against diabetes mellitus^15^. In 2022, Aboulthana *et* *al*^16^ emphasized that *Portulaca oleracea* leaf extracts showed a higher hepatoprotective effect when incorporated with zinc oxide nanoparticles (ZnO-NPs). Due to the toxicity induced by the ingestion of Ag-, Au-, and ZnO-NPs as proposed by Aboulthana *et al*.^17^, the present study was carried out to investigate the biological efficiency of selenium nanoparticles (Se-NPs) and copper oxide nanoparticles (CuO-NPs) biosynthesized using extracts isolated from *Sinapis alba* L. seeds.

## Materials and methods

### Preparation of different plant seeds extracts

White mustard (*S. alba*) seeds were purchased from a local herbal medicine supplier (Ragab El-Attar HerbalStore, Cairo, Egypt). A specimen of the seeds was authenticated by Dr. Gamal Farag, a taxonomist at the National Agriculture Center in Egypt. Subsequently, this specimen was deposited in the herbarium of the national research centre (NRC) in Cairo, Egypt, and Voucher No. M2280) was included in the methodology section. The powdered plant seeds (1 kg) were dried and successively extracted with petroleum ether (60–80 °C), methyl alcohol and distilled water using the cold extraction method. The extracts were then filtered and evaporated entirely using a rotary evaporator at 50 ^o^C. The three extracts obtained were separately filtered through a *Whatman No.* 1 filter paper and then concentrated in a rotary evaporator at 45 °C under reduced pressure until dryness. Furthermore, a portion of the air-dried seed powder underwent phytochemical screening to identify the different classes of chemical constituents responsible for the plant’s key biological activities.

### Phytochemical screening analysis

The carbohydrates and/or glycosides, flavonoids (free and combined), coumarins, saponins, alkaloids and/or nitrogenous compounds, sterols and/or triterpenes, tannins and anthraquinones (free and combined) were determined in P. ether, methanolic, and aqueous extracts using conventional methods.

### Metabolite profiling

#### Chromatographic analysis

The lipoidal constituents in the P. ether extract were investigated using gas chromatographic techniques coupled with a mass spectrometer (model Shimadzu GC/MS–QP5050A) on a Mass spectrometer (Agilent 6890) at 70 eV and Finnigan Mass spectrometer (Model 3200) at 70 eV. The constituents were identified by comparing their spectral fragmentation patterns with those in the available database libraries Wiley (Wiley Int.) USA and NIST (Nat. Inst. St. Technol., USA) and/or published data. The quantitative determination was performed by integrating the area under the peak.

The different phenolic and flavonoidal compounds were identified in the methanolic and aqueous extracts using high performance liquid chromatography (HPLC) (an Agilent 1260 series) in the Central labs. Net Unit at NRC, Cairo, Egypt. The separation was carried out using an Eclipse C18 column (4.6 mm x 250 mm i.d., 5 μm). The mobile phase consisted of water (A) and 0.05% trifluoroacetic acid in acetonitrile (B) at a flow rate of 0.9 ml/min. The mobile phase was programmed consecutively in a linear gradient as follows: 0 min (82% A); 0–5 min (80% A); 5–8 min (60% A); 8–12 min (60% A); 12–15 min (82% A); 15–16 min (82% A) and 16–20 min (82%A). The multi-wavelength detector was monitored at 280 nm. The injection volume was 5 µl for each of the sample solutions. The column temperature was maintained at 40 °C.

From the aqueous extract, the carbohydrates were isolated under acidified conditions according to Laidlow and Percival^18^. The nature was determined according to the methods demnstrated by Evans^19^. The mucilage hydrolysates were analyzed according to Gertz^20^ using gas liquid chromatography (GLC) (HP 6890) with a flame ionization detector at 270 °C. The analysis was performed using a ZB-1701 column (30 m × 0.25 m × 0.25 μm), 14%cyanopropyl phenyl methyl. The carrier gas was helium at a flow rate of 1.2 ml/min under a pressure of 10.6 psi and a velocity of 41 cm/s. The injector chamber temperature was − 250 °C. Quantitative determination was based on peak area measurement while qualitative identification was carried out by comparing the retention times of the peaks with those of the authentic sugars.

The proteins were isolated from the aqueous *S. alba* seeds extract using the technique published by El-Gengaihi *et al*.^21^. The total polypeptides were dialyzed using a dialysis membrane and then hydrolyzed to amino acids for investigation using the HPLC Pico-Tag method suggested by White *et al*.^22^.

The main flavonoids were isolated from the methanolic *S. alba* seed extract through a silica gel column. Dichloromethane was used for elution, gradually increasing the polarity with methanol. The obtained fractions were analyzed by thin-layer chromatography (TLC) using the ratio of ethyl acetate: methanol: water (6:1:0.5), visualized under UV light, and sprayed with ammonia, ferric chloride, and anisaldehyde-sulfuric acid. Positively responding spots were purified on TLC plates, yielding 4 pure compounds.

Compounds 1 and 2 were isolated from the dichloromethane column fraction (95 and 90%, respectively). Compound 3 was separated from the dichloromethane column fraction (80%). Additionally, compound 4 was obtained from the dichloromethane column fraction (75%). The isolated compounds were identified using different spectral analyses: FT-IR with a Perkin-Elmer 283 spectrophotometer (Germany), H^1^-NMR and^13^C-NMR analyses with JEOL EX-500 MHz and 300 MHz spectrometers, and MS analysis with a 3200 Finnigan Model spectrometer. Melting points were determined using Koffler’s heating stage microscope.

The methanolic and aqueous *S. alba* seeds extracts were analyzed using liquid chromatography–electrospray ionization–tandem mass spectrometry (LC-ESI-MS/MS) with an Exion LC AC system for separation and SCIEX Triple Quad 5500 + MS/MS system equipped with an electrospray ionization (ESI) for detection. The separation was performed with an Ascentis^®^ Express 90 Å C18 Column (2.1 × 150 mm, 2.7 μm). The mobile phases consisted of two eluents A: 5 mM ammonium formate pH 8 and B: acetonitrile (LC grade). The mobile phase gradient was programmed as follows: 5% B at 0–1 min, 5-100% B from 1 to 20 min, 100% B from 20 to 25 min, 5% at 25.01 min, and 5% from 25.01 to 30 min. The flow rate was 0.3 ml/min and the injection volume was 5 µl. For MS/MS analysis, negative ionization mode was applied with a scan (EMS-IDA-EPI) from 100 to 1000 Da for MS1 with the following parameters: curtain gas: 25 psi; IonSpray voltage: -4500; source temperature: 500 °C; ion source gas 1 & 2 were 45 psi and from 50 to 1000 Da for MS2 with a declustering potential: -80; collision energy: -35. Compounds’ identification was performed using MS-DIAL using Respect library.

#### Spectrophotometric analysis

In the different *S. alba* seeds extracts (P. ether, methanolic and aqueous), concentrations of total polyphenolic compounds, total condensed tannins and total flavonoid content were quantified in all prepared extracts using the methods suggested by Singleton and Rossi^23^, Broadhurst and Jones^24^, and Arvouet-Grand *et al*.^25^, respectively. The results were expressed as mg gallic acid/100 g, µg/mL, and mg quercetin/100 g, respectively.

#### *In vitro* biological activities

All the *in vitro* biological activities were evaluated in the different extracts and repeated three times.

#### Antioxidant and scavenging activity

The antioxidant activity, represented by total antioxidant capacity (TAC) and iron reducing power (IRP), was evaluated following the methods demonstrated by Mohammed^26^. The scavenging activity was assessed by calculating inhibition percentages (%) and median inhibitory concentration (IC_50_) of each tested extract against Diphenyl-2-picryl-hydrazyl (DPPH)^27^, 2,2’-azinobis-(3-ethylbenzothiazoline-6-sulfonic acid) (ABTS)^28^, and nitric oxide (NO) radicals^29^ using ascorbic acid as a standard.

#### Anti-diabetic activity

It was assayed by calculating inhibition percentages (Inhib. %) and the IC_50_ of α-amylase^30^ and α-glucosidase enzymes^31^ using acarbose as a standard drug.

#### Anti-acetylcholinesterase activity

The Inhib. % and IC_50_ of acetylcholinesterase (AChE) enzyme were assessed by calculating them, following the method described by Ellman *et al*.^32^, with donepezil used as the standard drug.

#### Anti-arthritic activity

It was evaluated by calculating Inhib. % and IC_50_ for protein denaturation^33^ and proteinase enzyme^34^ using diclofenac sodium as the standard drug.

#### Anti-inflammatory activity

It was evaluated by determining the Inhib. % and IC_50_ values of two isoenzymes, cyclooxygenase-1 (COX-1) and cyclooxygenase-2 (COX-2) (ovine/human)^35^, along with the 5-LOX enzyme (human recombinant)^36^, using commercially available kits.

#### Cytotoxic activity

It was determined by calculating the IC_50_ values against human hepatocellular (HEPG-2), colon carcinoma (CACO-2), and lung cancer (A549) cells and compared to normal human fibroblast (BJ-1) using the 3-(4,5-dimethylthiazol-2-yl)-2,5-diphenyl tetrazolium bromide (MTT) assay as suggested by Vichai and Kirtikara^37^. Additionally, the enzymatic activities of the different extracts against caspase-3 and Bcl-2 were evaluated in the studied cancer cells (HEPG-2, CACO-2, and A549 cells) following the techniques reported in the literature^38^.

### Preparation of metal *S. alba* seeds nano-extract

The different *S. alba* seeds extracts were used for the green biosynthesis of selenium nanoparticles (Se-NPs) using the method demonstrated by Salem *et al*.^39^. They proposed that each *S. alba* extract was added to Na_2_SeO_3_ (2 mM) in a flask, where a combination was prepared. The flask was incubated on a rotary shaker for 3 h in the dark to obtain a homogenous mixture. The biosynthesized Se-NPs were then centrifuged, dried, and stored at room temperature for further analyses.

The copper oxide nanoparticles (CuO-NPs) were biosynthesized using a concentrated anhydrous copper sulfate solution (1 mM) as a metal precursor based on the method suggested by Rani Verma and Khan^40^. Each *S. alba* extract was mixed with the metal solution in a ratio of 4:1. The biosynthesis of CuO-NPs was monitored by a color change. The biosynthesized CuO-NPs were dried at 80 °C after centrifuging the colloidal solution at 4000 rpm for 15 min.

### Characterization of the biosynthesized M-NPs

The spectra of the biosynthesized M-NPs were characterized using a Shimadzu UV-VIS recording spectrophotometer UV-240 after diluting the samples (10-fold) with deionized water at λ 200–800 nm. Their surface zeta potentials were measured using the laser zeta meter (Malvern Zetasizer 2000, Malvern, Worcestershire, UK) following the method demonstrated by Meléndrez and colleagues^41^ who suggested that the particles could be neutral (with a zeta potential between − 10 and + 10 mV), strongly cationic (greater than +30 mV) or strongly anionic (less than − 30 mV). Moreover, their average hydrodynamic sizes were determined using the laser diffraction method with multiple scattering techniques employing dynamic light scattering (DLS) (Malvern Zetasizer Nano ZS, Malvern Instruments Ltd., Malvern, United Kingdom)^42^.

### The median lethal doses (LD_50_)

The median lethal doses (LD_50_) of the various native extracts and metal nano-extracts were determined by counting the number of dead mice after 24 h of oral administration of high doses of each studied extract. The LD_50_ was calculated using the equation suggested by Paget and Barnes^43^.

## Results and discussion

### Phytochemical screening

The analysis of the dried residues resulted in the determination of the percentages along with the physical and chemical characteristics of the P. ether, methanolic, and aqueous extracts from *S. alba* seeds, which are outlined in (Supplementary Table 1). The oily P. ether extract contained mainly sterols and/or terpenes, while the methanolic and aqueous *S. alba* extracts contained carbohydrates, flavonoids, and tannins. The chemical characteristics of all studied extracts used for the biosynthesis of Se- and CuO-NPs showed that no obvious differences were identified between the native extracts and the biosynthesized metal nano-extracts. It was found that the aqueous *S. alba* extract responded positively to the carbohydrate test, and the amount of isolated carbohydrates was 63%. No gelatinous precipitate was produced after adding potassium hydroxide. On the contrary, ruthenium red formed a crimson color indicating the presence of mucilage rather than pectin. Eight free sugars were identified in the mucilage hydrolysate upon GLC investigation, accounting for 74.23% of the total hydrolysate’s weight. As shown in (Table 1), glucose (29.04%) was the predominant identified sugar, followed by galactose (17.05%), mannose (8.12%), arabinose (6.31%), xylose (4.72%), rhamnose (4.28%), ribose (3.58%), and mannitol (1.13%). The analysis’s outcome was consistent with Cui et al.^44^.

**Table 1:** GLC analysis of mucilage hydrolysate in the aqueous extract of *S. alba* L. Seeds.

Identified sugars	Rt (min.)	Relative percentage (%)
Xylose	16.7	4.72
Arabinose	17.0	6.31
Ribose	17.7	3.58
Rhamnose	18.8	4.28
Mannitol	21.5	1.13
Galactose	23.0	17.05
Mannose	23.2	8.12
Glucose	23.7	29.04
Total identified sugars		74.23

The aqueous *S. alba* extract contained 25% proteins. As presented in (Table 2), there were eighteen identified amino acids, consisting of 326.47 mg/g essential amino acids and 648.75 mg/g non-essential amino acids. It was established that threonine and methionine were the main essential amino acids, representing 74.38 mg/g (7.63%) and 56.17 mg/g (5.76%), respectively. However, histidine (98.63 mg/g, 10.11%), tyrosine (93.52 mg/g, 9.59%), and aspartic acid (92.84 mg/g, 9.52%) were the chief non-essential amino acids.

**Table 2:** Free amino acids in the aqueous extract of *S. alba* L. Seeds.

Essential amino acids	Conc. (mg/g)	Relative percentage in total amino acids	Non essential amino acids	Conc. (mg/g)	Relative percentage in total amino acids
Threonine	74.38	7.63	Aspartic acid	92.84	9.52
Valine	39.02	4.00	Glutamic acid	79.61	8.16
Methionine	56.17	5.76	Serine	88.54	9.08
Isoleucine	25.06	2.57	Glycine	35.19	3.61
Cysteine	23.29	2.39	Histidine	98.63	10.11
Leucine	17.34	1.78	Arginine	53.47	5.48
Phenyl alanine	35.81	3.67	Proline	58.91	6.04
Lysine	34.02	3.49	Alanine	48.04	4.93
Tryptophan	21.38	2.19	Tyrosine	93.52	9.59
Total	326.47	33.48	Total	648.75	66.52

As shown in (Supplementary Table 2), the constituents in the P. ether *S. alba* extract have been identified using gas chromatography coupled with a mass spectrometer by comparing their spectral fragmentation patterns with those in available database libraries. A total of 45 compounds were identified, representing 89.05% of the overall composition. The extracted oil was comprised of 11 saturated hydrocarbons (21.22%), 5 unsaturated hydrocarbons (15.39%), 3 fatty alcohols (4.51%), 3 aldehydes (6.37%), 7 fatty acids (15.91%), 13 esters (23.94%), and 3 monoterpenes (1.71%). n-Decane (3.95%) was the major identified saturated hydrocarbon, while 5-eicosene (5.72%) was the main unsaturated one. 1-Tridecanol (2.03%) and 2-heptenal (3.45%) were the principal identified fatty alcohols and aldehydes, respectively. Additionally, methyl palmitate and methyl linoleate (4.17 and 4.43%, respectively) were the chief detected esters. The identified monoterpenes consisted of mentha-6,8-diene, terpinyl acetate, and limonene with values of 0.29, 0.64, and 0.78%, respectively. This is consistent with the findings obtained by AbdulAlimMd *et al*.^45^.

Data depicted in Table 3 indicates that the methanolic and aqueous extracts of *S. alba* comprised 16 compounds, as identified through HPLC analysis focusing on phenolics and flavonoids. The primary phenolic compounds found in both extracts were chlorogenic acid and gallic acid, with concentrations of 1848.87 and 220.77 µg/g, respectively. Quercetin (168.06 µg/g) and hesperetin (121.62 µg/g) were the principal identified flavonoids in the methanolic and aqueous extracts, respectively. As illustrated in (Supplementary Figs. 1 and 2), the HPLC chromatograms of the methanolic and aqueous *S. alba* extracts were compared to the standard phenolics and flavonoids (Supplementary Fig. 3).

**Table 3:** HPLC analysis of the phenolics and flavonoids in methanolic and aqueous *S. alba* seeds extracts.

Compound	Methanolic extract			Aqueous extract		
	Area	Conc. (µg/mL = 20 mg/mL)	Conc. (µg/g)	Area	Conc. (µg/mL = 20 mg/mL)	Conc. (µg/g)
Gallic acid	229.65	19.83	991.52	51.13	4.42	220.77
Chlorogenic acid	270.02	36.98	1848.87	31.98	4.38	218.96
Catechin	16.33	4.04	202.18	4.19	1.04	51.87
Methyl gallate	190.41	10.39	519.66	15.94	0.87	43.51
Coffeic acid	50.71	3.90	194.99	7.52	0.58	28.93
Syringic acid	174.27	11.82	590.85	14.36	0.97	48.70
Rutin	2.20	0.25	12.75	11.55	1.34	67.06
Ellagic acid	32.23	5.97	298.71	7.02	1.30	65.06
Coumaric acid	28.03	0.88	44.21	6.53	0.21	10.30
Vanillin	103.81	4.54	227.18	4.09	0.18	8.96
Ferulic acid	0.00	0.00	0.00	1.80	0.12	6.15
Naringenin	8.35	1.01	50.36	1.10	0.13	6.66
Daidzein	3.65	0.23	11.26	3.29	0.20	10.17
Quercetin	24.40	3.36	168.06	2.89	0.40	19.93
Cinnamic acid	79.08	1.46	72.84	15.06	0.28	13.87
Apigenin	0.00	0.00	0.00	0.00	0.00	0.00
Kaempferol	24.23	1.88	93.93	0.00	0.00	0.00
Hesperetin	4.68	0.27	13.65	41.73	2.43	121.62

### Characterization of the isolated flavonoids

**Compound 1** was isolated as yellow amorphous powder with R_*f*_ 0.73, UV: λmax, nm: (MeOH) 240 (sh), 269, 345 which are characteristic for flavonoids (+ NaOMe): 268, 337 (sh), 405 directing for polyhydroxylation, (+ AlCl_3_): 266 (sh), 275 (sh), 389descriptive for presence of OH group at C-5, (+ AlCl_3_ + HCl): 263 (sh), 278 (sh), 355, 386, (+ NaOAc): 281, 345 (sh), 417, (+ NaOAc + Boric acid): 272, 320 (sh), 346no bathchromic shift indicative of absence of *ortho*-dihydroxyl groups. ESI-MS exhibited molecular weight at *m/z* 300 calculated for molecular formula C_16_H_12_C_6_, in addition toother mass fragments at *m/z* 240, 225, 136, 121, 119, 105, 91, 79, 57, and 43. ^1^H-NMR (400 MHz, CD_3_OD, δppm): 6.85 (1 H, s, H-3), 6.19 (1 H, s, H-6), 6.48 (1 H, s, H-8), 7.61 (1 H, d, J = 2.0, H-2`), 6.88 (1 H, d, J = 8.2, H-5`), 7.53 (1 H, dd, J = 2.0, 8.2,H6`), 4.02 (3 H, s, O-CH_3_)^13^. C-NMR (125 MHz, CD_3_OD, δppm): 165.4 (C-2), 102.8 (C-3), 179.0 (C-4), 159.2 (C-5), 98.6 (C-6), 162.5 (C-7), 92.8 (C-8), 156.4 (C-9), 103.2 (C-10), 118.5 (C-1`), 109.3 (C-2`), 137.4 (C-3`), 145.6 (C-4`), 120.6 (C-5`), 116.3 (C-6`), and 55.6 (O-CH3). From the spectral outcomes and through reviewing literature, the isolated compound was structurally elucidated as chrysoeriol (3’-methoxy derivative of luteolin)^46,47^.

**Compound 2** was isolated as yellow amorphous powder with R_*f*_ 0.68, UV: λmax, nm: (MeOH) 237 (sh), 255, 340 distinguishing for flavonoids (+ NaOMe): 263,415 guiding for existence of polyhydroxygroups, (+ AlCl_3_): 235 (sh), 250, 337no bathochromic shift suggestive for absence of free hydroxyl group in C-5, (+ AlCl3 + HCl): 236 (sh), 353, 334, (+ NaOAc): 252, 336, (+ NaOAc + Boric acid): 258, 340.EI-MS spectrum showed molecular weight at *m/z* = 312 corresponding to molecular formula as C_18_H_16_O_5_, beside to other significant fragments at *m/z* 265, 237, 209, 151, 135, 107. ^1^H-NMR (400 MHz, CD_3_OD, δppm): 6.63 (1 H, s, H-3), 6.25 (1 H, d,J = 2.4, H-6), 6.48 (1 H, d, J = 2.4, H-8), 7.81 (1 H, d, H-2′),7.32 (1 H, d, H-3′), 7.45 (1 H, d, 9.0, H-5′),7.74 (1 H, d, 8.5, H-6′), 3.82 (3 H, s, 5-OCH3), 3.89 (3 H, s, 7-OCH3), 3.76 (3 H, s, 4′-OCH3)^13^. C-NMR (125 MHz, CD_3_OD, δppm): 158.6 (C-2), 108.5 (C-3),180.4 (C-4), 166.4 (C-5), 99.7 (C-6), 164.3 (C-7), 94.7 (C-8), 157.8 (C-9), 107.9 (C-10), 122.6 (C-1′), 125.4 (C-2′), 117.6 (C-3′), 136.5 (C-4′), 129.1 (C-5′),126.4 (C-6′), 56.7 (5-OCH3), 55.8 (7-OCH3), 55.3 (4′-OCH3). The isolated compound was identified as 5, 7, 4′-Trimethylapigeninbased on the spectral data and literature survey^48^.

**Compound 3** was isolated as white powder with R_*f*_ 0.79, melting point 197, UV: λmax, nm: (MeOH) 254, 263 sh, 368 corresponded to flavonol nucleus^46^; (+ NaOMe): 257, 368, 412 directorial for polyhydroxylation. (+ AlCl_3_): 269,274sh, 372,431 showing bathochromic shift suggestive of *ortho*-dihydroxylation.(+ AlCl_3_ + HCl): 268,273sh, 432 stable UV dataapprovingpresence of OH group at C-5. (MeOH + NaOAc): 267, 289sh, 366, 427sh, (+ NaOAc + Boric acid): 269, 375, 438 showed bathochromic shift pinpoint for the presence of 3`, 4`dihydroxy group. ESI-MS analysis presented molecular weight at *m/z* = 464 equivalent to molecular formula C_21_H_20_O_12_, beside to *m/z* 318 corresponded to myricetinaglycone (C_15_H_10_O_8_) after rhamnose loss and *m/z* 303, 287, 271, 179, 133. The obtained data were matched with earlier studies^49,50^. ^1^H-NMR (400 MHz, CD_3_OD, δppm): 6.31 (1 H, d, J = 1.8, H-6), 6.43 (1 H, d, J = 1.8, H-8), 7.02 (1 H, s, H-2`, H-6`),5.24 (1 H, s, H-1``),3.29 (1 H, m, H-2″), 3.24(1 H, m, H-3″), 3.19 (1 H, d, H-4″), 3.14 (1 H, dd, H-5″), 3.09 (3 H, d, H-6″)^13^. C-NMR (125 MHz, CD_3_OD, δppm): 155.8 (C-2), 134.2 (C-3), 176.9 (C-4), 159.8 (C-5), 99.4 (C-6), 163.9 (C-7), 92.6 (C-8), 154.2 (C-9), 103.5 (C-10), 120.6 (C-1`), 107.8 (C-2`, C-6`), 143.1 (C-3`, C-5`), 137.2 (C-4`), 102.4 (C-1``), 69.4 (C-2``), 70.8 (C-3``), 72.4 (C-4``), 68.1 (C-5``), 19.0 (C-6``). The data provided was corroborated with previously documented by El-Kashak et al.^51^. and Sobeh et al.^52^. Hence, the structure was confirmed to be myricetin3-*O*-rhamnoside, which was confirmed through the analysis of the methanolic *S. alba* extract using LC-ESI-MS/MS.

**Compound 4** was isolated as yellow crystals with R_*f*_ 0.64, melting point 185,UV: λmax, nm: (MeOH) 253, 269sh, 354 giving value greater than 350 nm indicating for flavonol nucleus; (+ NaOMe): 258, 301sh, 367 guiding for polyhydroxylation. (+ AlCl_3_): 267,277sh, 369,425 with bathochromic shift indicative of hydroxylation at C-5 or *ortho*-dihydroxyl groups; (+ AlCl_3_ + HCl): 266, 278 sh, 423 stable UV values confirmingexistence of OH group at C-5. MeOH + NaOAc (263,287 sh, 361, 413 sh, (+ NaOAc + Boric acid): 266, 442 with bathochromic shift of band I guides the presence of 3`, 4`dihydroxy group. ESI-MS spectrum showed molecular weight at *m/z* = 594 corresponding to molecular formula as C_27_H_30_O_15_, beside to other significant fragments at *m/z* 448 for quercetinrhamnoside, 302 for quercetinaglycone, 287, 164, 129. ^1^H-NMR (400 MHz, CD_3_OD, δppm): 6.37 (1 H, d, J = 2.3, H-6), 6.58 (1 H, d, J = 2.3, H-8), 7.25 (1 H, d, J = 2.1, H-2′), 6.93 (1 H, d, J = 7.4, H-5′), 7.18 (1 H, dd, J = 2.1, 7.4, H-6′), 5.19 (1 H, s, H1″), 5.27 (1 H, s, H-1′″), 3.76 (1 H, m, H-2′″), 3.59 (1 H, m, H-2″), 3.50 (1 H, dd, H-3′″), 3.46 (1 H, m, H-3″), 3.28 (1 H, m, H-4′″),3.22 (1 H, d, H-4″), 3.18 (1 H, m, H5′″), 3.12 (1 H, dd, H-5″), 1.25 (3 H, d, H-6′″), 1.18 (3 H, d, H-6″)^13^. C-NMR (125 MHz, CD_3_OD, δppm): 157.1 (C-2), 140.1 (C-3),182.3 (C-4), 176.4 (C-5), 102.1 (C-6), 159.6 (C-7), 96.1 (C-8), 151.3 (C-9), 103.5 (C-10), 119.7 (C-1′), 113.7 (C-2′), 141.3 (C-3′), 144.0 (C-4′),114.3 (C-5′),118.6 (C-6′), 98.5 (C-1″), 95.1 (C-1′″), 67.9 (C-2″), 67.2 (C-2′″),68.7 (C-3″), 68.2 (C3′″), 69.8 (C-4″), 69.4 (C-4′″),66.9 (C-5″), 66.5 (C-5′″), 18. 7 (C-6″), 18.1 (C-6′″). Through the comparison of spectroscopic records with scientific findings^47,53^, it was determined to be quercetin 3,7-*O*-dirhamnoside. This conclusion was supported by the analysis of the methanolic extract using LC-ESI-MS/MS.

### Phytochemical constituency profiling

LC-ESI-MS/MS analysis was conducted to investigate the presence of important phytochemical constituents in the various *S. alba* seed metal nano-extracts. The MS analysis was performed using ESI in negative ion mode, known for its high sensitivity in detecting different classes of phenolic compounds^54^. During the analysis, the most intense peak corresponding to the deprotonated molecular ion [M–H]^−^ obtained in the ESI-MS spectrum was further subjected to mass fragmentation. The tentative characterization of the compounds was carried out based on the deprotonated [M–H]^−^ molecular ion and the fragments produced. The identity of each compound was confirmed by searching against the online mass spectral database and by comparing the data with that published in the literature^55^.

The identification of various phenolic compounds, such as phenolic acids, flavonoids (both aglycones and glycosides), and glucosinolates, was achieved through LC-ESI-MS/MS analysis of the methanolic and aqueous *S. alba* extracts. This analysis was conducted on both Se and CuO- *S. alba* nano-extracts. Data from this assay provide a comprehensive summary of all the identified compounds, including their retention time (Rt), molecular ion [M-H]-, molecular formula, and MS/MS fragment ions.

As shown in Supplementary Table 3, the methanolic *S. alba* extract contained 7 phenolic acids (protocatechuic acid, gallic acid, caffeic acid, chlorogenic acid, sinapinic acid, quinic acid, and ferulic acid). Caffeic acid, sinapinic acid, and ferulic acid and have been previously identified by Rasera et al.^56^. and Khatib and Al-Makky^57^. Additionally, 9 flavonoid aglycones (luteolin, naringenin, gallocatechin, epicatechin, limocitrin, kaempferol, quercetin, isorhamntin, sinensetin) and 8 flavonoid glycosides (apigenin 7-*O*-glucoside, kaempferol 3-*O*-glucoside, isoquercetin, isorhamnetin 3-*O*-rutinoside, quercetin 3,7-*O*-dirhamnoside, rutin, myricetin 3-*O*-rhamnoside, and myricetin7-*O*-glucoside) were characterized through the present investigation. Previous studies reported the presence of kaempferol, quercetin, and rutin in *S. alba* seeds. Moreover, naringenin and epicatechin have been identified by Jiang *et al*.^58^. and Martinović *et al*.^59^.

As shown in Supplementary Table 4, the aqueous extract contained 8 phenolic acid derivatives (ferulic acid, 3,4-dimethoxy cinnamic acid, ellagic acid, methyl gallate, hydroxymethoxy benzoic acid, caffeoylhexoside, hydroxydimethoxy benzoic acid, *p*-coumaroylquinic acid), and 7 flavonoid glycosides (apigenin 7-*O*- glucoside, kaempferol-3-*O*-diglucoside, kaempferol 3-*O*-xyloside, kaempferol 3-*O*-rutinoside, vitexin-2’’-*O*-rhamnoside, quercetin 3-*O*-arabinoside, luteolin-8-*C*-β-D-glucopyranoside 7-*O*-rhamnoside) which were identified in *S. alba* seeds for the first time in the current study, except kaempferol-3-diglucoside, which was previously identified by Jiang *et al*.^58^. Furthermore, 4 aliphatic glucosinolates were characterized in the aqueous extract (sinigrin, gluconapin, progoitrin, glucobrassicanapin), in addition to 3 aromatic aryl glucosinolates (glucotropaeolin, gluconasturtiin, glucosinalbin), and three sulfur-containing glucosinolates (glucoerucin, glucoibervirin, glucoiberin), which were previously characterized^60,61^.

As sulfur-containing glycosides produced from amino acids with alkyl or aryl substitution in the side chain, glucosinolates are sulfur-rich secondary metabolites^62^. Their distinct properties and breakdown products, known as isothiocyanates, contribute to the characteristic flavor and bitter taste of mustard seeds^63^. According to the amino acid precursors, glucosinolates can be divided into two categories: aromatic glucosinolates, which come from phenylalanine and tyrosine, and aliphatic glucosinolates, which come from alanine, leucine, isoleucine, valine, and methionine^64^. Glucosinolates have a variety of biological properties due to their diverse side chains. They work as strong anti-inflammatory agents by preventing the synthesis of mediators linked to inflammation. Additionally, they have exceptional antioxidant qualities by scavenging and removing reactive oxygen species, which reduces their negative effects^62,65^. Furthermore, they demonstrate significant anticancer effects by impeding the entry of carcinogens into target sites and activating crucial hepatic enzymes that combat various types of carcinogens^66^.

### *In vitro *biological activities

#### The major active phyto-constituents

Data depicted in Table 4 show that the major phyto-constituents (total polyphenols, total condensed tannins, and total flavonoids) in the different *S. alba* seed extracts (P. ether, methanolic and aqueous) utilized for biosynthesis in metal *S. alba* seeds nano-extracts. It was found that the methanolic extract contains the highest concentrations of total polyphenols (133.34 ± 0.31 mg gallic acid/100 g), condensed tannins (53.34 ± 0.12 µg/mL) and total flavonoid compounds (66.67 ± 0.16 mg quercetin/100 g), followed by the aqueous extract and then the P. ether extract. Our findings are in accordance with Stojanovi´c *et al*.^67^. and are consequently supported by Montaner et al.^68^. Concentrations of these phyto-constituents decreased in the P. ether extract that was used for the biosynthesis of Se-NPs (106.67 ± 0.25 mg gallic acid/100 g, 42.62 ± 0.78 µg/mL, and 42.67 ± 0.10 mg quercetin/100 g, respectively). Also, they decreased highly in the extract used for the biosynthesis of CuO-NPs (96.97 ± 0.23 mg gallic acid/100 g, 36.27 ± 0.08 µg/mL, and 38.79 ± 0.09 mg quercetin/100 g, respectively). This is consistent with the study proposed by Gunti *et al*.^69^. , who found that some of these constituents are consumed for the reduction and stabilization of metal ions during the phyto-synthesis of M-NPs.

**Table 4:**
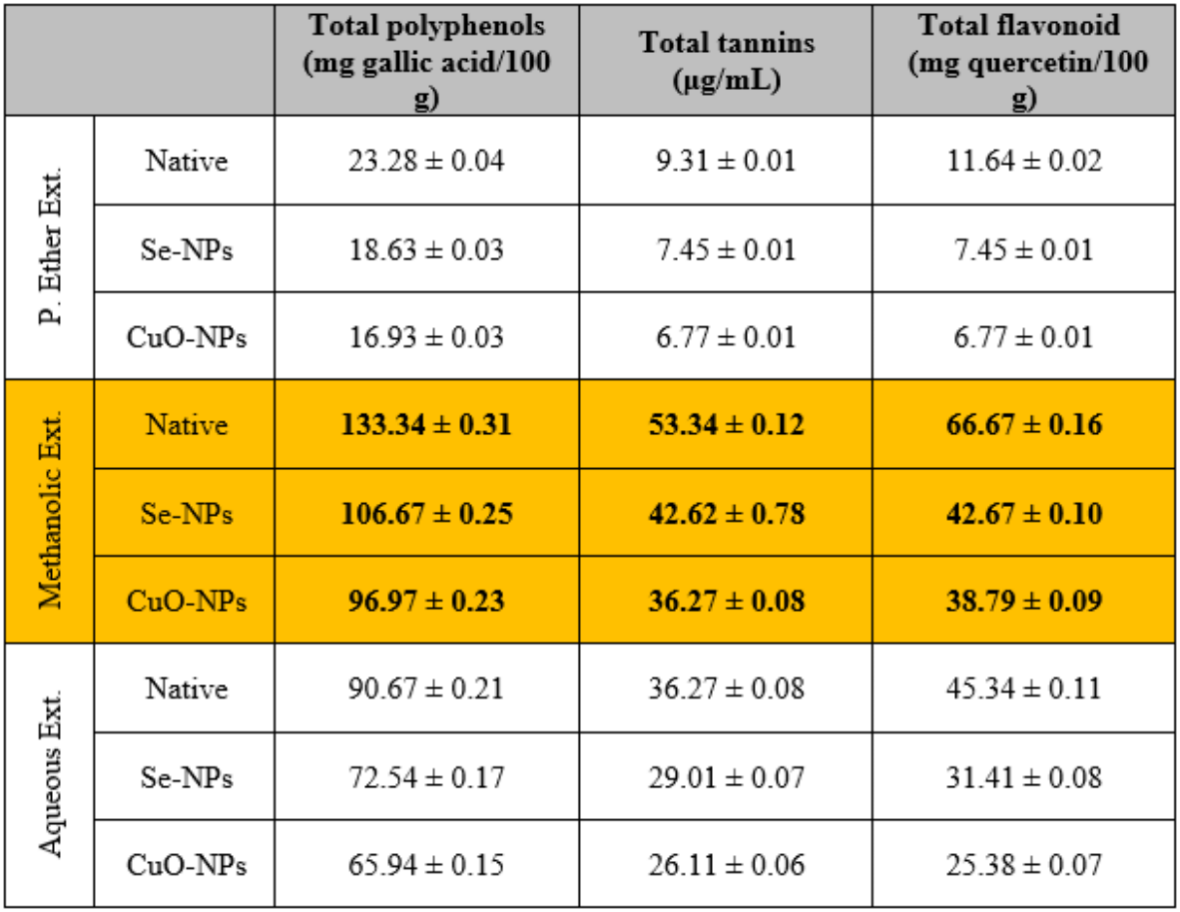
Concentrations of the major active phyto-constituents in different *S. alba* seeds metal nano-extracts and compared to the native extracts. The values were calculated from *n* = 3/extract and given as mean ± SE. The orange cell indicates the extract with the highest concentrations of the phyto-constituents.

#### Antioxidant and scavenging activity

Data presented in Table 5 shows that the *in vitro* antioxidant activity of different *S. alba* seed extracts was assessed by measuring TAC and IRP in addition to their scavenging activities against DPPH and ABTS radicals. The activity against DPPH and ABTS radicals was expressed as IC_50_ values, where a low IC_50_ value indicates strong antioxidant activity. Among all the studied extracts, the methanolic extract exhibited the highest antioxidant activity and had the greatest inhibitory effect against DPPH (52.55 ± 0.12%) and ABTS radicals (76.19 ± 0.18%), followed by the aqueous extract and then the petroleum ether extract at equal concentrations (100 µg/mL). Among all the studied extracts, the methanolic extract exhibited the highest activity. This finding is consistent with Özcan *et al*.^70^. This may be attributed to the redox properties of the phenolic compounds enabling them to donate hydrogen during the biosynthesis process and, therefore, be used as reducing agents^54^. At the same concentration, the standard ascorbic acid inhibited the DPPH and ABTS radicals by 61.70 ± 0.12 and 72.78 ± 0.04%, respectively. In terms of IC_50_ values, the methanolic extract had the lowest IC_50_ against DPPH (4.22 ± 0.01 µg/mL) and ABTS radicals (3.18 ± 0.01 µg/mL) compared to the other studied extracts.

**Table 5:**
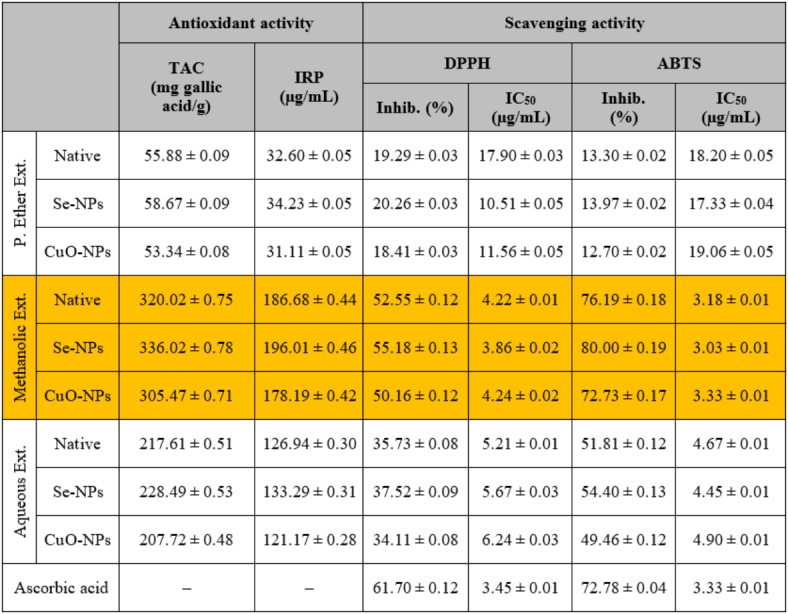
The *in vitro* antioxidant and radicals scavenging activities of different *S. alba* seeds metal nano-extracts and compared to the native extracts. The values were calculated from *n* = 3/extract and given as mean ± SE. The orange cell indicates the most effective extract.

The antioxidant activity (TAC and IRP) increased slightly in the methanolic extract used for the biosynthesis of Se-NPs (336.02 ± 0.78 mg gallic acid/g and 196.01 ± 0.46 µg/mL, respectively) and decreased slightly in the extract used for the biosynthesis of CuO-NPs (305.47 ± 0.71 mg gallic acid/g and 178.19 ± 0.42 µg/mL, respectively) compared to the native methanolic extract itself. This is consistent with the findings obtained by Ikram *et al*.^71^, who postulated that Se-NPs derived from secondary metabolites have stronger antioxidant properties due to the presence of functional groups on their surfaces. Therefore, plant-based Se-NPs may be added as promising therapeutic agents that play an effective role in treating diseases caused by oxidative stress and the overproduction of reactive species.

The scavenging activity increased slightly in the methanolic extract used for the biosynthesis of Se-NPs against DPPH (Inhib. 55.18 ± 0.13%; IC_50_ 3.86 ± 0.02 µg/mL) and ABTS radicals (Inhib. 80.00 ± 0.19%; IC_50_ 3.03 ± 0.01 µg/mL) and decreased slightly in the extract used for the biosynthesis of CuO-NPs against DPPH (Inhib. 50.16 ± 0.12%; IC_50_ 4.24 ± 0.02 µg/mL) and ABTS radicals (Inhib. 72.73 ± 0.17%; IC_50_ 3.33 ± 0.01 µg/mL) compared to the native methanolic extract itself. This is consistent with Alam *et al*.^72^. who proposed that the phyto-synthesized Se-NPs displayed strong antioxidant and scavenging activities, which are dependent not only on size but also on functional moieties on the surfaces of the M-NPs that are occupied by secondary metabolites. According to Puri *et al*.^73^, biosynthesized Se-NPs with smaller diameters had greater scavenging activity because they had more free radical reactive sites, which improved their antioxidant capacity in a size-dependent manner.

#### Anti-diabetic activity

Both α-amylase and α-glucosidase play an effective role in regulating blood glucose levels due to their catalytic effects, which stimulate the breakdown of carbohydrates into monosaccharides^74^. The α-amylase and α-glucosidase inhibitory assays are considered measurements of anti-diabetic activity, which were then compared to acarbose (standard drug)^75^. During the present study, it was observed that the total methanolic extract possessed the highest inhibitory effect on α-amylase activity (Inhib. 45.72 ± 0.11%; IC_50_ 4.75 ± 0.02 µg/mL) and α-glucosidase (Inhib. 33.17 ± 0.11%; IC_50_ 3.82 ± 0.01 µg/mL) compared to the other extracts (Table 6). This finding is consistent with Kifle *et al*.^76^, who demonstrated that the α-amylase inhibitory activity might be related to the presence of phenolic compounds, which have an inhibitory effect on the α-amylase enzyme. The lowest inhibitory effect on both enzymes was noticed with the P. ether extract compared to the standard acarbose, which exhibited inhibitory effects at the same concentration on α-amylase activity (Inhib. 68.27 ± 0.02%; IC_50_ 3.18 ± 0.01 µg/mL) and α-glucosidase (Inhib. 55.39 ± 0.01%; IC_50_ 2.29 ± 0.01 µg/mL).

**Table 6:**
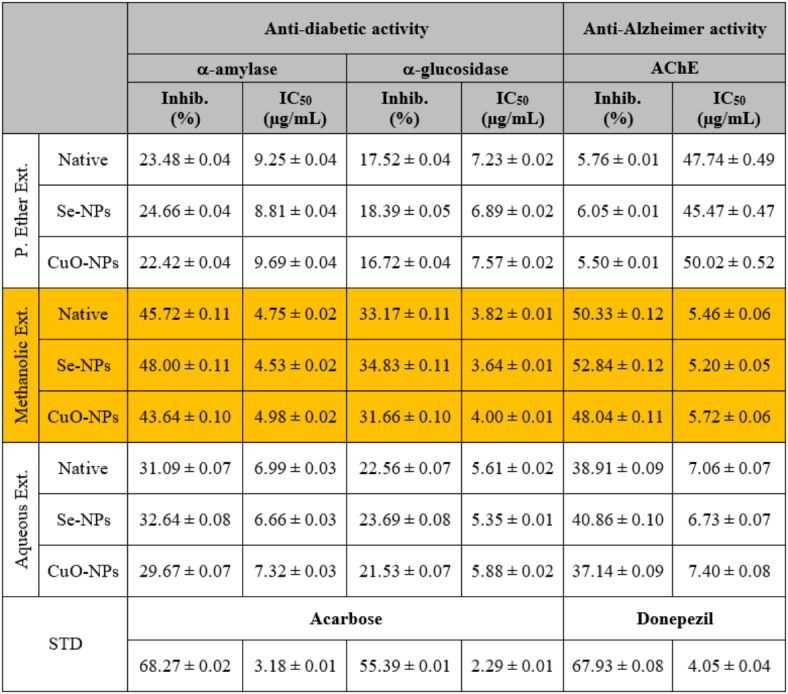
The *in vitro* anti-diabetic and anti-Alzheimer activities of different *S. alba* seeds metal nano-extracts and compared to the native extracts. The values were calculated from *n* = 3/extract and given as mean ± SE. The orange cell indicates the most effective extract.

The inhibitory effect slightly increased in the methanolic extract used for the biosynthesis of Se-NPs against the activity of α-amylase (Inhib. 48.00 ± 0.11%; IC_50_ 4.53 ± 0.02 µg/mL) and α-glucosidase enzyme (Inhib. 34.83 ± 0.11%; IC_50_ 3.64 ± 0.01 µg/mL) and decreased slightly in the extract used for the biosynthesis of CuO-NPs (Inhib. 43.64 ± 0.10; IC_50_ 4.98 ± 0.02 µg/mL and 31.66 ± 0.10%; IC_50_ 4.00 ± 0.01 µg/mL, respectively) compared to the native methanolic extract itself. Our findings agreed with Kainat *et al*.^77^, who emphasized that the hydrolytic enzymes, especially α-amylase, were the most susceptible diabetic enzyme to be interacted with the M-NPs. The phyto-synthesized Se-NPs displayed dose-dependent inhibition of carbohydrate hydrolyzing enzymes based on the molecular interactions between the enzyme and the Se-NPs, such as van der Waals, electrostatic forces, hydrogen, and hydrophobic bonds. Further studies are required to determine the mode of inhibition (competitive or non-competitive)^78^. Deepa *et al*.^79^. reported that the inhibitory effects of the biosynthesized Se-NPs could be attributed to van der Waals forces, which act on the nanosized particles, reducing the carbohydrate-hydrolyzing enzyme. This is more likely to lead to non-competitive inhibition of the enzyme activity.

#### Anti-acetylcholinesterase activity

It is responsible for the hydrolysis of acetylcholine (ACh) in the cholinergic synapses associated with β-amyloid plaques. Hence, the inhibition of AChE is a therapeutic strategy for Alzheimer’s disease management^80^. During the present experiment, it was observed that the total methanolic extract exhibited the highest inhibitory effect on AChE activity (Inhib. 50.33 ± 0.12%; IC_50_ 5.46 ± 0.06 µg/mL), followed by the aqueous extract (Inhib. 38.91 ± 0.09%; IC_50_ 7.06 ± 0.07 µg/mL) and then the P. ether extract (Inhib. 5.76 ± 0.01%; IC_50_ 47.74 ± 0.49 µg/mL) (Table 6). In comparison, at the same concentration, the standard donepezil inhibited the activity of the AChE enzyme by 67.93 ± 0.08% (IC_50_ 4.05 ± 0.04 µg/mL). This is consistent with Taslimi *et al*.^81^, who reported that the anti-acetylcholinesterase activity is strongly attributed to the presence of total phenolics and flavonoids, which possess antioxidant and anti-diabetic activities.

The inhibitory effect increased slightly in the methanolic extract used for the biosynthesis of Se-NPs against the activity of AChE enzyme (Inhib. 52.84 ± 0.12%; IC_50_ 5.20 ± 0.05 µg/mL) and decreased slightly in the extract used for the biosynthesis of CuO-NPs (Inhib. 48.04 ± 0.11%; IC_50_ 5.72 ± 0.06 µg/mL) compared to the native methanolic extract itself. The biosynthesized Se-NPs have a higher ability to inhibit the activity of the AChE enzyme due to the degradation of aromatic amino acid residues and/or an increase in the number of water molecules in the hydration shell of the protein portion, leading to a change in the refractive index^82^.

#### Anti-arthritic activity

The denaturation of proteins and the activity of the proteinase enzyme are responsible for the arthritic reaction. The ability of the extract to inhibit protein denaturation and the proteinase enzyme indicates its potential for anti-inflammatory activity. Therefore, the anti-arthritic activity was assessed by measuring these parameters^83^. The current study showed that both the methanolic and aqueous extracts exhibited the same inhibitory effect on protein denaturation (Inhib. 35.12 ± 0.08 and 35.08 ± 0.08%, respectively) and proteinase enzyme (Inhib. 31.43 ± 0.10; IC_50_ 8.72 ± 0.04 µg/mL and 31.46 ± 0.08%; IC_50_ 8.78 ± 0.03 µg/mL, respectively). This may refer to the presence of phytoconstituents including alkaloids, flavonoids, carbohydrates, glycosides, saponins, and tannins, in addition to phenolic compounds, which are well-known for their anti-arthritic activity as demonstrated by Chandur *et al*.^84^. and consequently supported by Hussien *et al*.^85^. The P. ether extract showed the lowest inhibitory effect compared to the standard diclofenac sodium that possessed an inhibitory effect at the same concentration on protein denaturation (Inhib. 46.45 ± 0.02%) and proteinase enzyme (Inhib. 44.60 ± 0.01%; IC_50_ 6.18 ± 0.02 µg/mL) (Table 7). The biosynthesized Se-NPs and CuO-NPs using all studied *S. alba* extracts showed no inhibitory changes in protein denaturation and the activity of the proteinase enzyme compared to their native extracts indicating that the presence of Se- and CuO-NPs caused no effect on these measurements.

**Table 7:**
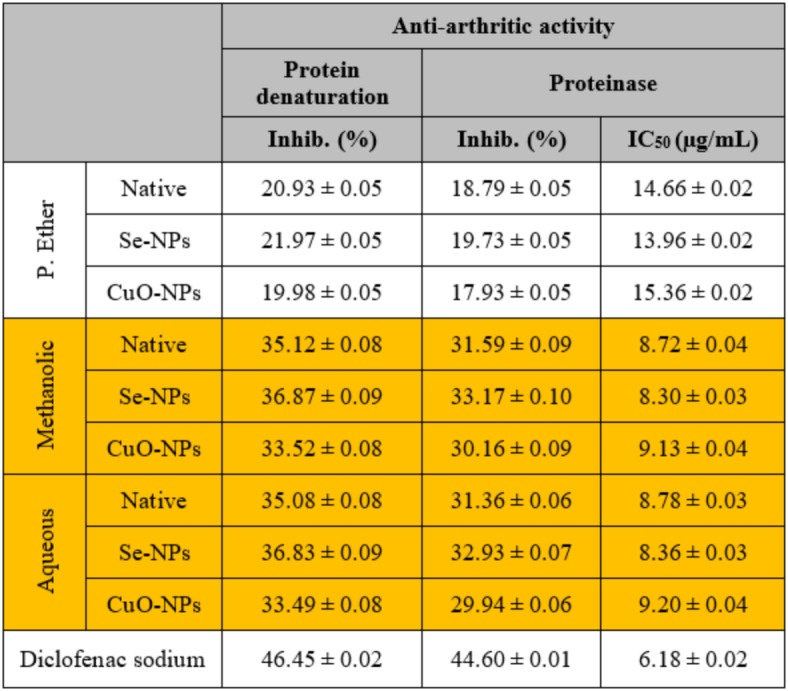
The *in vitro* anti-arthritic activity of different *S. alba* seeds metal nano-extracts and compared to the native extracts. The values were calculated from *n* = 3/extract and given as mean ± SE. The orange cell indicates the most effective extract.

#### Anti-inflammatory activity

The lipoxygenase (5-LOX) and cyclooxygenase (COX) are well-known pro-inflammatory enzymes that are frequently used to evaluate the anti-inflammatory activity of metal nano-extracts^86^. COX is characterized by two isoforms, COX-1 and COX-2, which differ in their intracellular locations and chemical structures, but they perform the same function^87^.

During the present study, it was observed that the total methanolic extract exhibited the highest inhibitory effect on the activities of COX-1 (Inhib. 66.92 ± 0.21%; IC_50_ 5.71 ± 0.03 µg/mL), COX-2 (Inhib. 69.17 ± 0.21%; IC_50_ 4.70 ± 0.02 µg/mL), and 5-LOX enzymes (Inhib. 54.02 ± 0.21%; IC_50_ 7.75 ± 0.05 µg/mL) compared to the other studied extracts (Table 8).

**Table 8:**
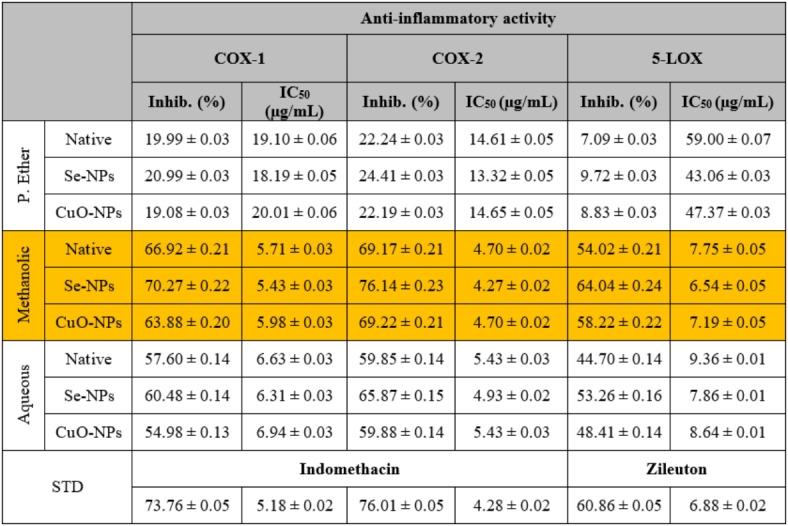
The *in vitro* anti-inflammatory activities of different *S. alba* seeds metal nano-extracts and compared to the native extracts. The values were calculated from *n* = 3/extract and given as mean ± SE. The orange cell indicates the most effective extract.

Although natural products show promising potential as treatments for inflammatory conditions, researchers face many limitations in fully understanding their mechanism of action. This might be attributed to the complexity of natural products, which makes it difficult to identify which active compounds are responsible for the anti-inflammatory effects. Moreover, it is difficult to predict the probable side effects caused by interactions of the active constituents with other drugs and medications^88^. From another point of view, the methanolic extract possessed its anti-inflammatory activity due to the binding of phenolic compounds at the lobby region and hydrophobic region of the COX binding site of the COX enzyme through hydrophobic interactions^89^.

The P. ether extract showed the lowest inhibitory effect on the activities of these enzymes compared to the standards that possessed an inhibitory effect at the same concentration on COX-1 (Inhib. 73.76 ± 0.05%; IC_50_ 5.18 ± 0.02 µg/mL), COX-2 (Inhib. 76.01 ± 0.05%; IC_50_ 4.28 ± 0.02 µg/mL), and 5-LOX (Inhib. 60.86 ± 0.05%; IC_50_ 6.88 ± 0.02 µg/mL).

The inhibitory effect increased slightly in the methanolic extract used for the biosynthesis of Se-NPs against the activities of COX-1 (Inhib. 70.27 ± 0.22%; IC_50_ 5.43 ± 0.03 µg/mL), COX-2 (Inhib. 76.14 ± 0.23%; IC_50_ 4.27 ± 0.02 µg/mL), and 5-LOX enzymes (Inhib. 64.04 ± 0.24%; IC_50_ 6.54 ± 0.05 µg/mL) and decreased slightly in the extract used for the biosynthesis of CuO-NPs (Inhib. 63.88 ± 0.20%; IC_50_ 5.98 ± 0.03 µg/mL, Inhib. 69.22 ± 0.21%; IC_50_ 4.70 ± 0.02 µg/mL, and Inhib. 58.22 ± 0.22%; IC_50_ 7.19 ± 0.05 µg/mL, respectively) compared to the native methanolic extract itself. This agrees with Rehman *et al*.^90^, who proposed that the M-NPs exhibited anti-inflammatory activity by blocking COX-1, COX-2, and 5-LOX in a concentration dependent manner. Furthermore, these enzymes might be inhibited due to their denaturation on M-NPs surface and/or charge effects. Cha *et al*.^91^. added that the smaller M-NPs might be expected to have the strongest inhibitory effects. The inhibition percentage depends on the shape of the biosynthesized M-NPs. The inhibition rate in enzyme activity was observed to increase with increasing concentrations of nanopyramids and nanoplates, while the enzyme activity was found to be virtually unchanged for all concentrations of nanospheres.

#### Cytotoxic activity

Cancer is a group of diseases characterized by malignant neoplasms arising from uncontrolled cell proliferation that invade and destroy the surrounding tissue leading to death if not controlled^92^. During the present study, it was found that the methanolic extract exhibited higher cytotoxic activity against HEPG-2, CACO-2, A549, and BJ-1 cells with the lowest IC_50_ values (304.30 ± 0.11, 202.60 ± 0.17, 560.50 ± 0.13, and 182.00 ± 0.11 µg/mL, respectively) compared to the other studied extracts (Table 9). This may be related to the presence of various bioactive compounds, mainly tannins and flavonoids, which have demonstrated antiproliferative and cytotoxic activities by inducing apoptosis, impeding cell growth, and perturbing the progression of the cell cycle in cancer cells^93^. Moreover, the presence of these bioactive constituents possesses cytotoxic activity by intercalating with DNA and causing inhibition of DNA topoisomerases^94^.

**Table 9:**
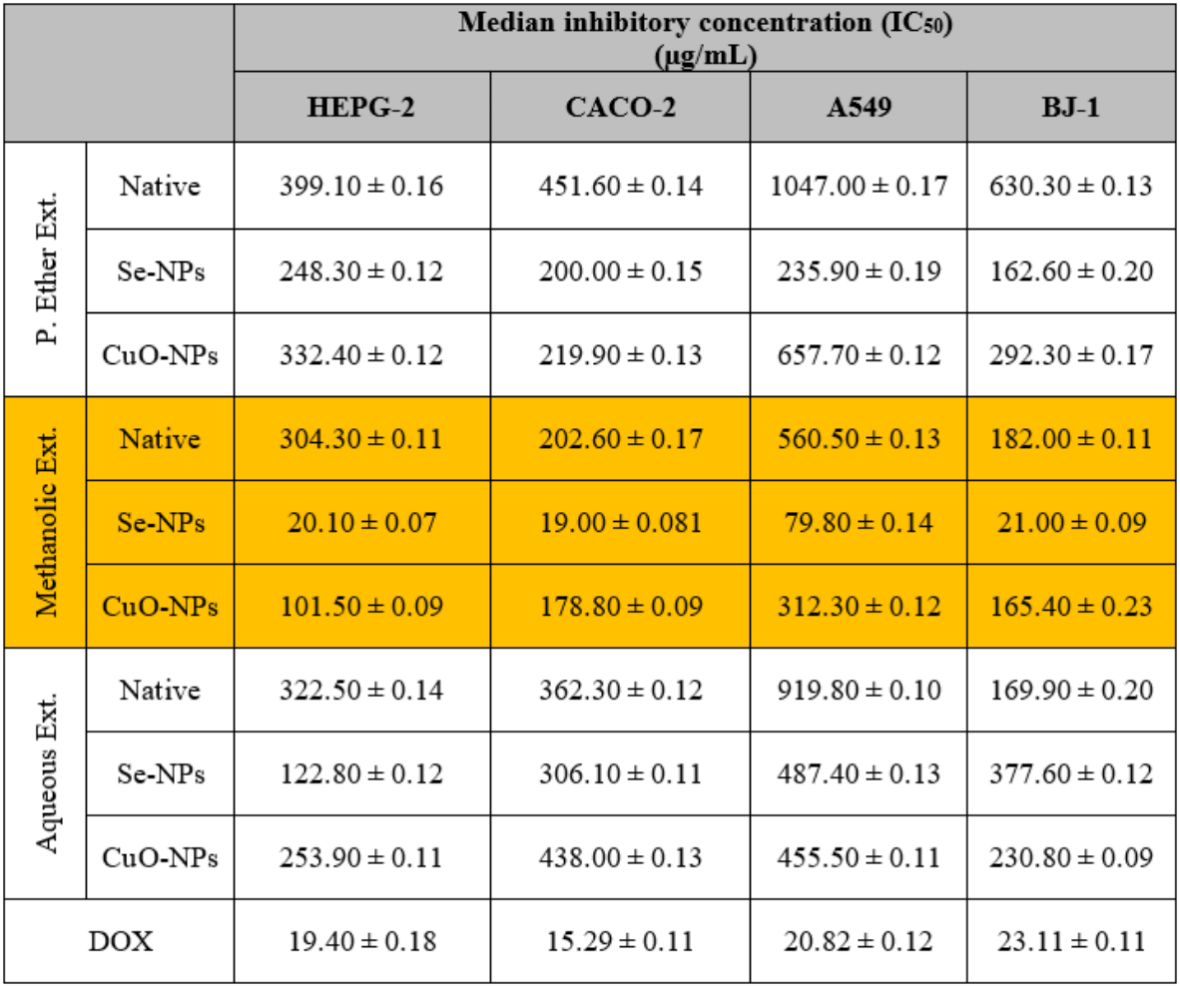
The *in vitro* cytotoxic activities of different *S. alba* seeds metal nano-extracts against human hepatocellular carcinoma (HEPG-2), colon (CACO-2), and lung (A549) cancer cell lines and compared to the normal human fibroblast cells (BJ-1). The values were calculated from *n* = 3/extract and given as mean ± SE. The orange cell indicates the most effective extract.

The P. ether extract showed the lowest cytotoxic activity (399.10 ± 0.16, 451.60 ± 0.14, 1047.00 ± 0.17, and 630.30 ± 0.13 µg/mL, respectively) compared to the standard that possessed the highest cytotoxic activity (19.40 ± 0.18, 15.29 ± 0.11, 20.82 ± 0.12, and 23.11 ± 0.11 µg/mL, respectively).

The cytotoxic activity increased highly in the methanolic extract used for the biosynthesis of Se-NPs (20.10 ± 0.07, 19.00 ± 0.08, 79.80 ± 0.14, and 21.00 ± 0.09 µg/mL, respectively) and slightly in the extract used for the biosynthesis of CuO-NPs (101.50 ± 0.09, 178.80 ± 0.09, 312.30 ± 0.12, and 165.40 ± 0.23 µg/mL, respectively) compared to the native methanolic extract itself. This finding is consistent with Tripathi et al.^95^. , who demonstrated that the biosynthesized Se-NPs induce apoptotic cancer cell death and suppress cell proliferation and survival after being internalized through endocytosis in a concentration dependent manner. Wang et al.^96^. reported that there is a relationship between phyto-synthesized Se-NPs and the cell cycle’s lifespan. They enhance anticancer activity due to their ability to conjugate with siRNA. Furthermore, due to the diminutive dimensions and expansive surface area of the M-NPs, they have the ability to inhibit antioxidant molecules and hence generate excessive ROS in tumor tissues, thus inducing oxidative stress. This can lead to infiltrating cells, activation of apoptosis, and inflammatory pathways^97^.

#### Enzymatic assay

The common anti-cancer therapeutic techniques involve stimulating proapoptotic molecules and blocking anti-apoptotic ones^98^. As suggested by Naglah et al.^99^, the extract with cytotoxic activity increased the activity of the caspase-3 enzyme while decreasing the Bcl-2 level in the treated cancer cells compared to the untreated ones.

**Fig. 1:**
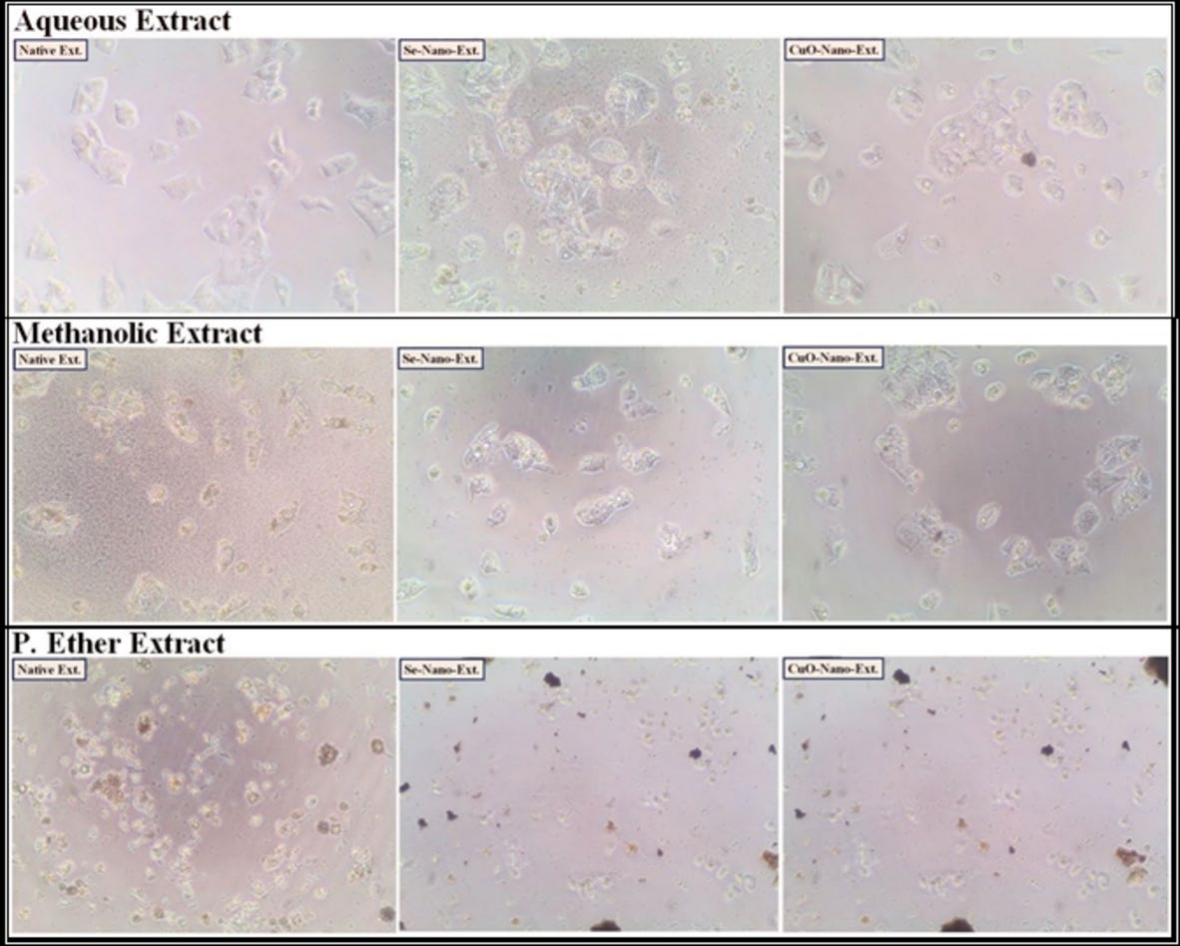
The human hepatocellular carcinoma (HEPG-2) cancer cell lines treated with the median inhibitory concentration (IC_50_) of different *S. alba* seeds metal nano-extracts.

**Fig. 2:**
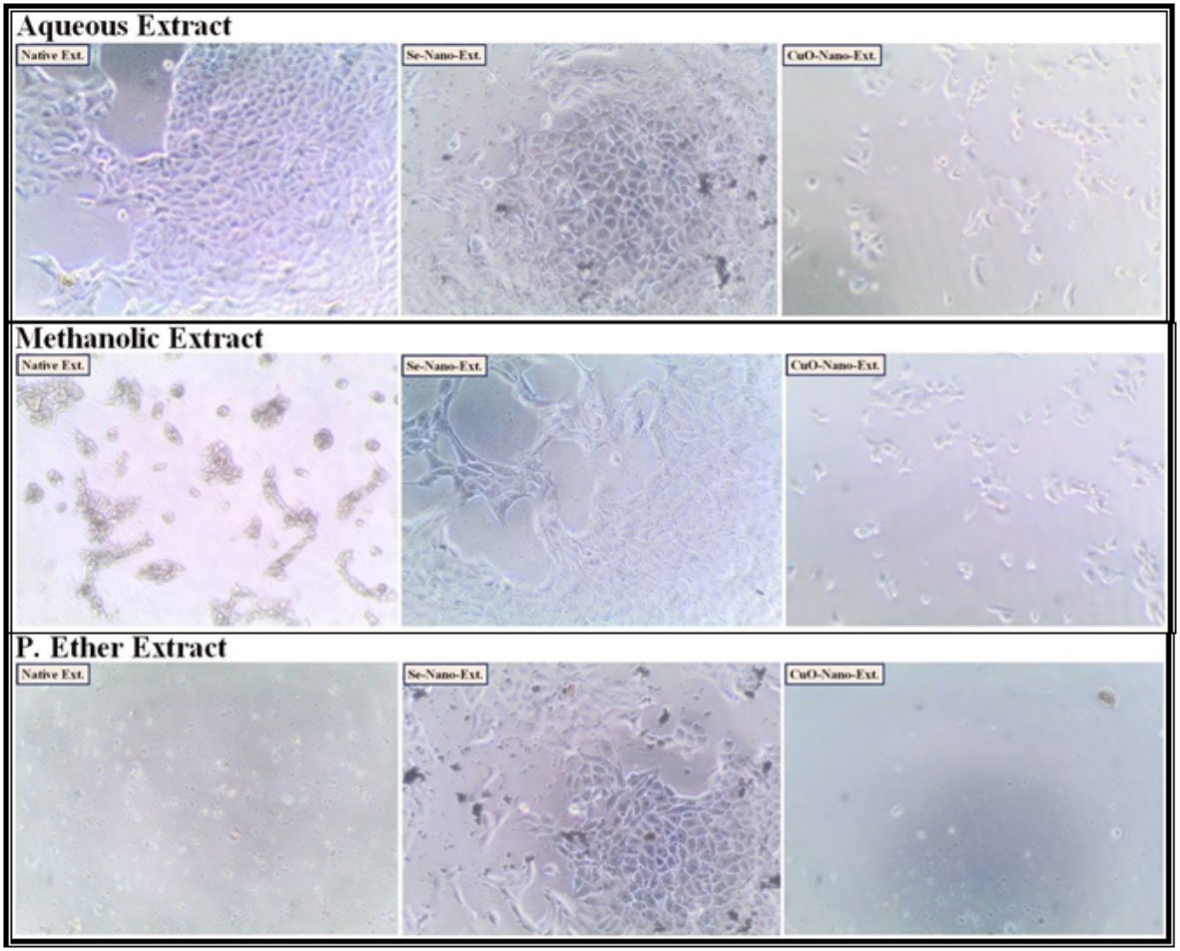
The human colon (CACO-2) cancer cell lines treated with the median inhibitory concentration (IC_50_) of different *S. alba* seeds metal nano-extracts.

**Fig. 3:**
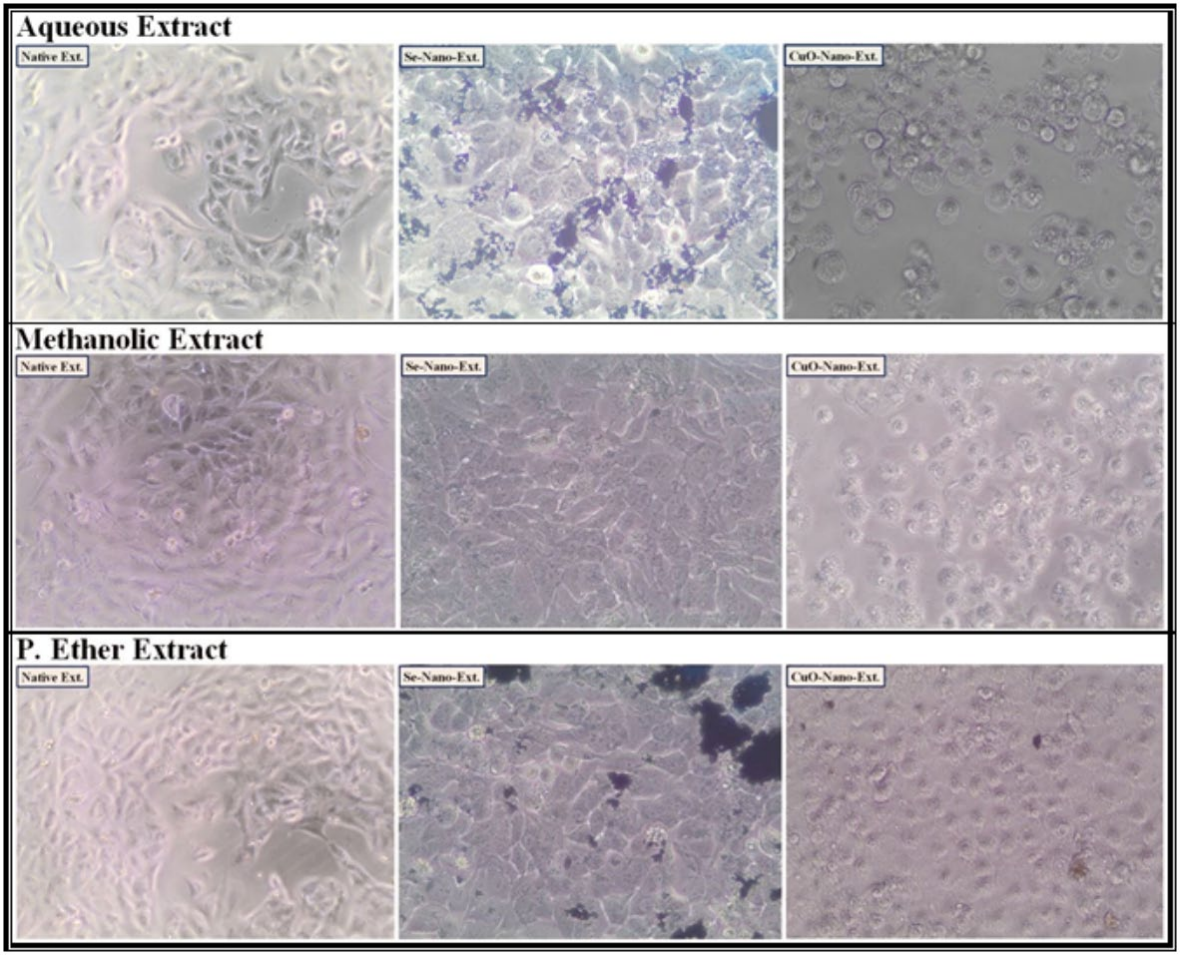
The human lung (A549) cancer cell lines treated with the median inhibitory concentration (IC_50_) of different *S. alba* seeds metal nano-extracts.

**Fig. 4:**
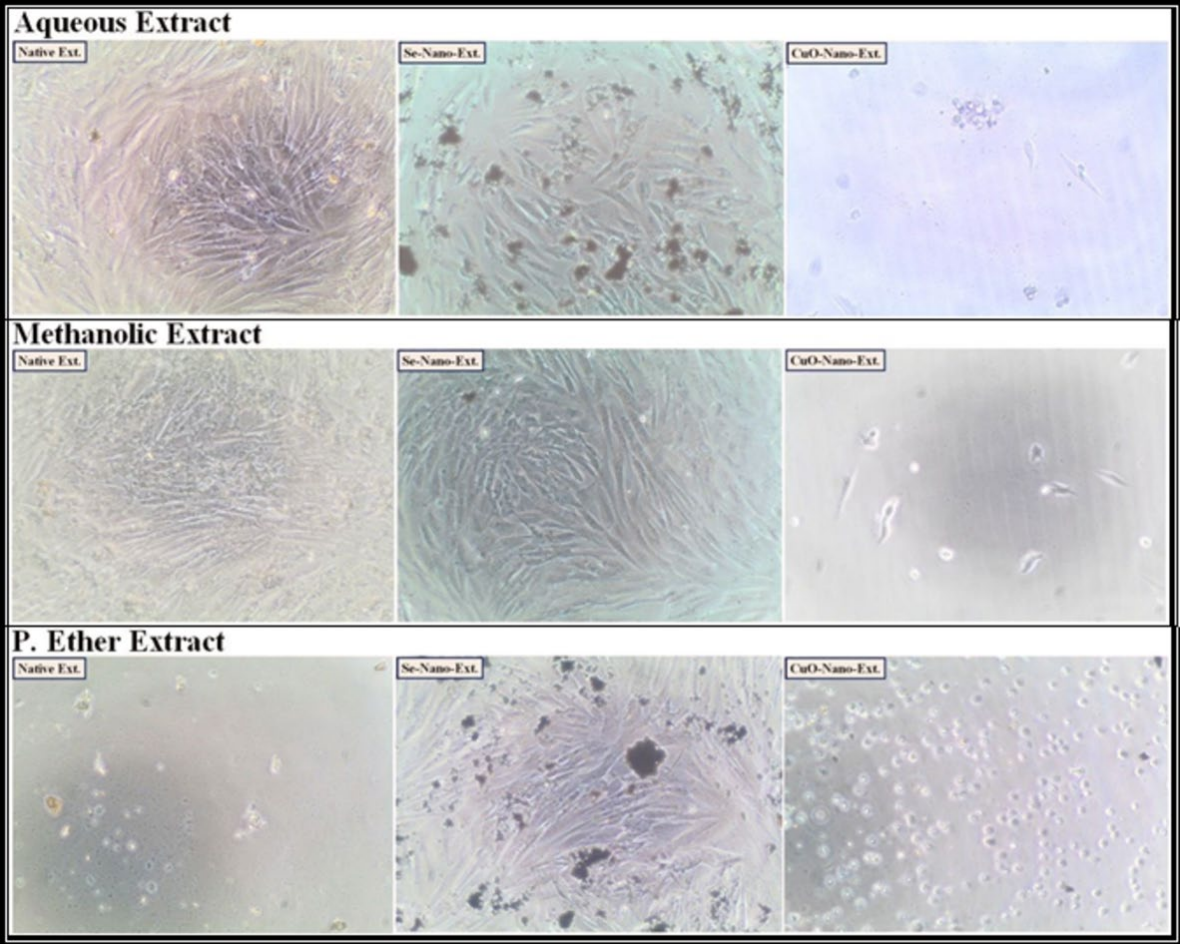
The normal human fibroblast cells (BJ-1) treated with the median inhibitory concentration (IC_50_) of different *S. alba* seeds metal nano-extracts.

After treating the HEPG-2, CACO-2, A549, and BJ-1 cells with the different studied *S. alba* seed extracts at IC_50_ values as presented in (Figs. 1, 2, 3 and 4), the activities of the caspase-3 and Bcl-2 enzymes were assayed in the treated cells. During the present study, the highest activity of the caspase-3 enzyme was observed in HEPG-2 (269.61 ± 2.10 Pg/mL), CACO-2 (234.96 ± 0.35 Pg/mL), A549 (219.76 ± 0.30 Pg/mL), and BJ-1 cells (213.34 ± 0.30 Pg/mL) treated with the methanolic extract, with the lowest activity of the Bcl-2 enzyme (4.31 ± 0.06, 2.22 ± 0.03, 4.86 ± 0.02, and 3.90 ± 0.02 ng/mL, respectively) observed in these treated cells (Tables 10 and 11). This is in accordance with Choi *et al*.^100^. who emphasized that the bioactive constituents in the methanolic extract stimulate the apoptotic pathway by up-regulating caspase-3 enzyme and down-regulating Bcl-2. Safari *et al*.^101^. added that the phytochemicals demonstrated significant cytotoxic activity against a wide range of cancer cells due to their ability to induce apoptosis in cancer cells through the regulation of caspases. Furthermore, they exhibit their cytotoxic potential through their anti-Bcl-2 activity by deactivating the Bcl-2 protein to bind with pro-apoptotic proteins, thereby stimulating apoptosis in cancer cells^102^. The lowest activity of the caspase-3 enzyme (184.47 ± 1.44, 193.74 ± 0.29, 181.20 ± 0.25, and 175.91 ± 0.25 Pg/mL, respectively) with the highest activity of the Bcl-2 enzyme (5.87 ± 0.08, 2.85 ± 0.04, 6.25 ± 0.02, and 5.02 ± 0.02 ng/mL, respectively) was observed in the cancer cells treated with the P. ether extract.

**Table 10:**
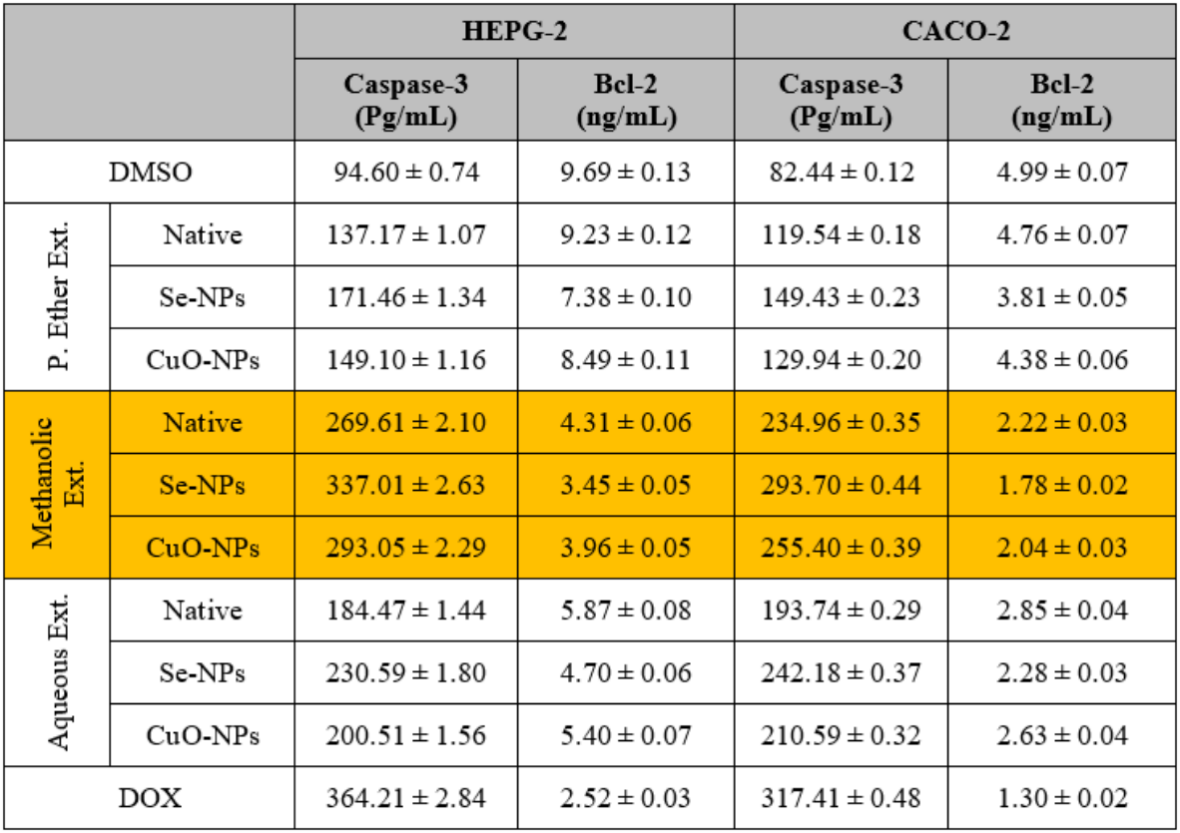
The enzymatic assay values after the treatment of human hepatocellular carcinoma (HEPG-2) and colon (CACO-2) cancer cells with different *S. alba* seeds metal nano-extracts and compared to the native extracts. The values were calculated from *n* = 3/extract and given as mean ± SE. The orange cell indicates the most effective extract.

**Table 11:**
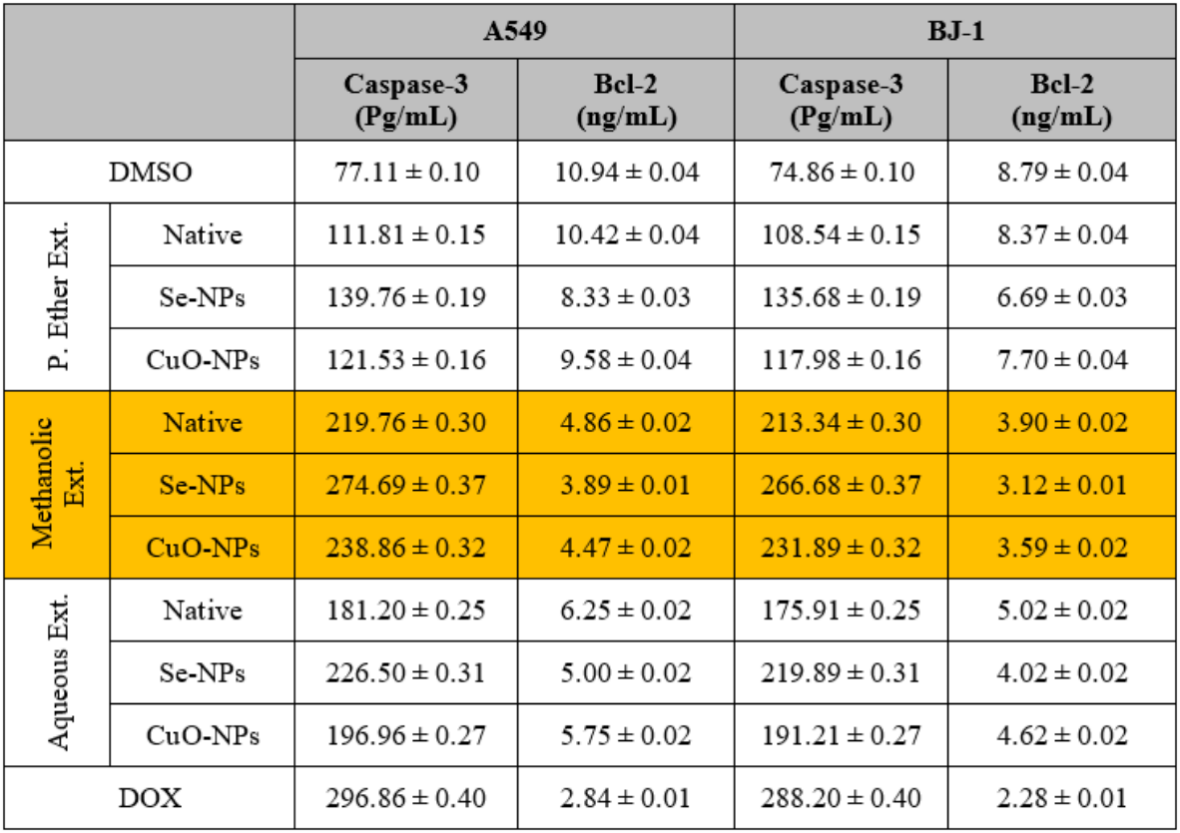
The enzymatic assay values after the treatment of human lung (A549) cancer cells and normal human fibroblast cells (BJ-1) and compared to the native extracts. The values were calculated from *n* = 3/extract and given as mean ± SE. The orange cell indicates the most effective extract.

The activity of the caspase-3 enzyme increased highly with a decrease in the Bcl-2 level in the cancer cells treated with the methanolic extract used for the biosynthesis of Se-NPs. Additionally, the activity of the caspase-3 enzyme increased slightly with a decrease in the Bcl-2 level in the cancer cells treated with the methanolic extract used for the biosynthesis of CuO-NPs compared to the untreated cells or the cells treated with the native methanolic extract itself. This is consistent with Khaled *et al*.^103^, who suggested that the biosynthesized M-NPs exert a good antitumor effect against the cancer cells by up-regulating the activity of the caspase-3 enzyme, along with down-regulating Bcl-2 levels, which consequently leads to inducing apoptosis. Furthermore, this up-regulation leads to depleting the expression of cyclin D1 and CDK-2 mRNA, and hence stimulates the arrest of the G1 cell cycle. Othman *et al*.^104^. added that the biosynthesized M-NPs induce apoptosis through the inhibition of the expression of the PI3K/AKT and Ras/Ras/ERK protein signaling pathways.

### Characterization of the biosynthesized metal nanoparticles

**Fig. 5:**
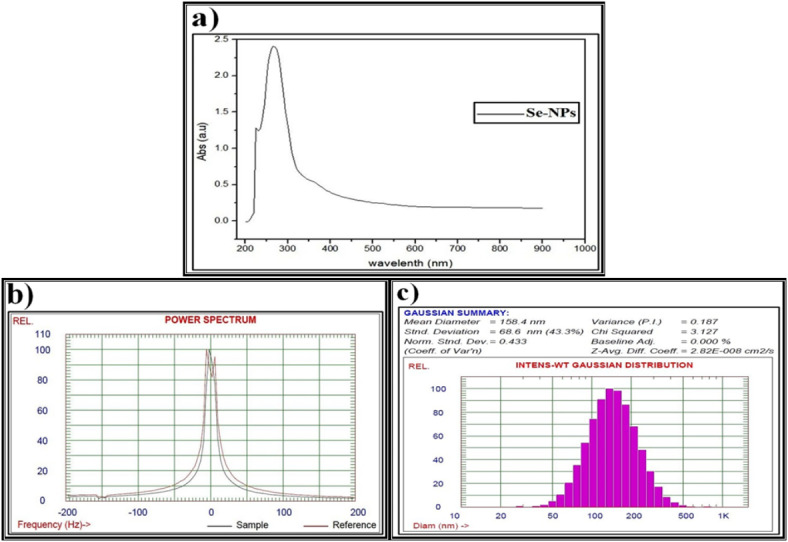
Data of the biosynthesized selenium nanparticles (Se-NPs) using petroleum ether *S. alba* seeds extract and characterized by (**a**) Ultraviolet-visible (UV-VIS) spectroscopy, (**b**) Zeta potential, (**c**) Dynamic light scattering (DLS).

As illustrated in Fig. 5a, the prepared Se-NPs, which were biosynthesized by P. ether *S. alba* seeds extract, showed a sharp peak at 295 nm, revealing the formation of Se-NPs. This agrees with El Lateef Gharib *et al*.^105^. who reported that the absorption peak of the typical surface plasmon was noticed at 296 nm in the UV-Vis spectrum, verifying the synthesis of nanosized sodium selenate. Furthermore, changing the color of the reaction mixture from uncolored to reddish confirms the biosynthesis of Se-NPs, which is represented by the characteristic peak between 200 and 400 nm and the red-shift increases with increasing particle sizes, as suggested by Anu *et al*.^106^.

The stability of M-NPs suspensions can be evaluated by the value of zeta potential, which is considered to indicate very good stability due to electrostatic interactions between the M-NPs and low molecular weight surfactants. Zeta potential values of only 20 mV or less can provide good stabilization due to the presence of sufficient repulsive forces^107^. During the current study, the zeta potential pattern confirmed the formation of the biosynthesized Se-NPs (Fig. 5b).

The average particle size determined by DLS analysis is influenced by the homogeneity percentages and plant metabolites that coat the surfaces of the M-NPs, leading to interference with the calculation^108^. It is widely recognized that the particle size measured using this technique tends to be slightly larger than the nominal size, possibly due to the measurement of a hydrodynamic size rather than a physical size^109^. The current study revealed that the particle size distribution of the synthesized Se-NPs has a primary diameter of around 150 nm (Fig. 5c). Additionally, the DLS results indicated a uniform droplet size distribution, and the Polydispersity Index confirmed the homogeneity of the droplet size of the biosynthesized Se-NPs. This finding is consistent with Nowruzi *et al*.^110^, who showed that a higher dispersion value corresponds to less uniformity in droplet size.

**Fig. 6:**
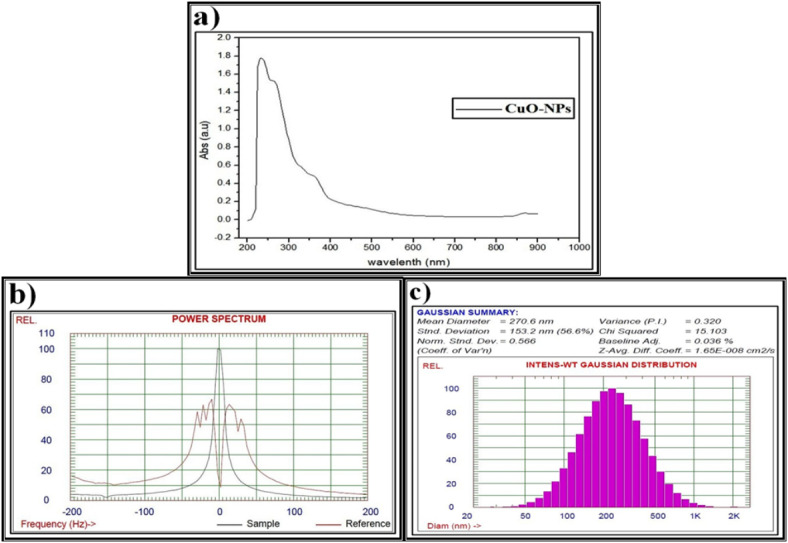
Data of the biosynthesized copper oxide nanoparticles (CuO-NPs) using petroleum ether *S. alba* seeds extract and characterized by (**a**) Ultraviolet-visible (UV-VIS) spectroscopy, (**b**) Zeta potential, (**c**) Dynamic light scattering (DLS).

The CuO-NPs which were biosynthesized by P. ether *S. alba* seeds extract were confirmed by one absorption peak at 255 nm and another weak but broad resonance centered peak at about 670 nm, indicating the formation of the biosynthesized CuO-NPs (Fig. 6a). This is in agreement with Akintelu *et al*.^111^, who proposed that the CuO-NPs formed between 200 and 350 nm.

The biosynthesized CuO-NPs have a zeta potential of 63.2 mV with a particle size around 100 nm, showing excellent stability (Fig. 6b). This is consistent with Kolahalam *et al*.^112^, who postulated that excellent physical colloidal stability is indicated by the zeta potential with a more positive or negative charge, which may be attributed to electrostatic repulsions between particles. The lower magnitude of the zeta potential may indicate the accumulation of particles.

Data from the DLS pattern reveal that the particle size distribution of the fabricated CuO-NPs has a main diameter around 200 nm (Fig. 6c). It shows that the particles were larger and polydisperse compared to those observed in the UV and zeta potential values. This agrees with Nzilu *et al*.^113^, who emphasized that the diameter of the biosynthesized CuO-NPs was in the range of 0 to 200 nm, with a large population. The value of the polydispersity index indicates that the individual size distribution was monodispersed due to agglomeration or aggregation during the biosynthesis process.

**Fig. 7:**
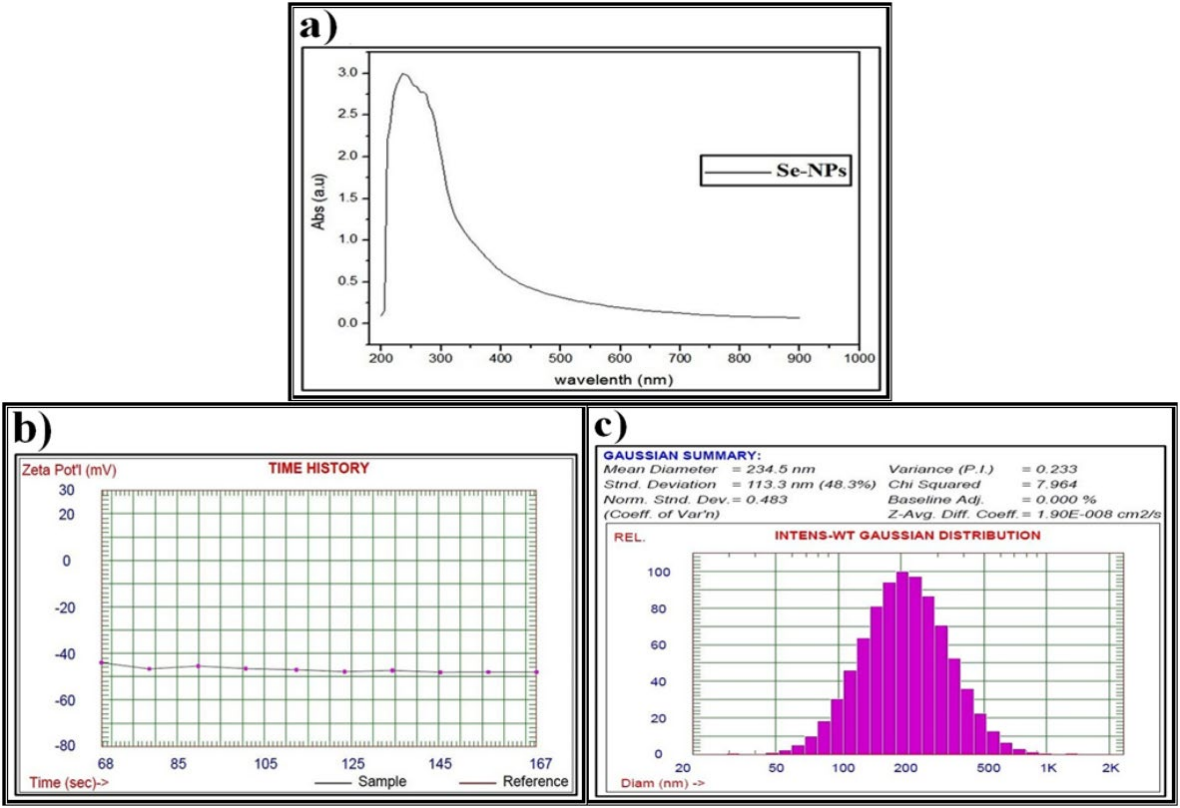
Data of the biosynthesized selenium nanparticles (Se-NPs) using methanolic *S. alba* seeds extract and characterized by (**a**) Ultraviolet-visible (UV-VIS) spectroscopy, (**b**) Zeta potential, (**c**) Dynamic light scattering (DLS).

**Fig. 8:**
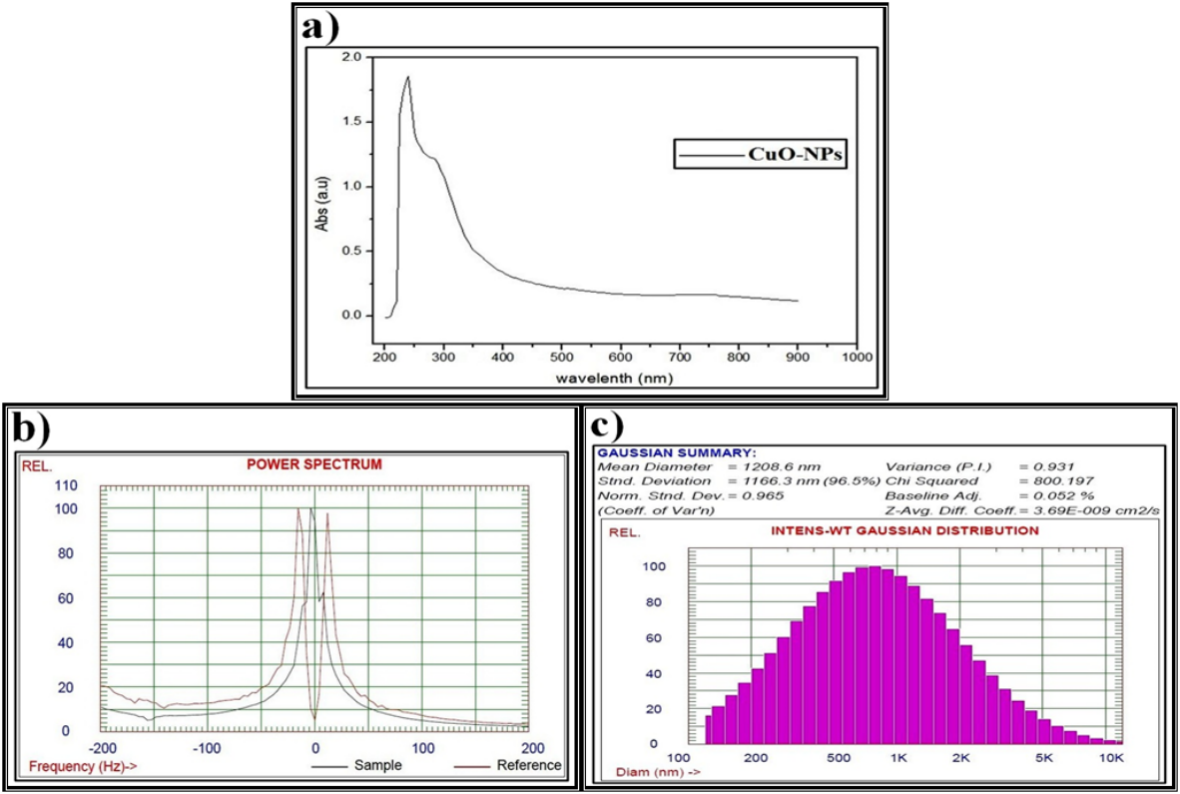
Data of the biosynthesized copper oxide nanoparticles (CuO-NPs) using methanolic *S. alba* seeds extract and characterized by (**a**) Ultraviolet-visible (UV-VIS) spectroscopy, (**b**) Zeta potential, (**c**) Dynamic light scattering (DLS).

The data obtained from the UV spectra, zeta potential, and DLS pattern confirm the formation of Se-NPs biosynthesized using methanolic *S. alba* seeds extract (Fig. 7) as discussed above. As illustrated in (Fig. 8), the UV spectra, zeta potential, and DLS pattern confirm the CuO-NPs biosynthesized using methanolic *S. alba* seeds extract.

**Fig. 9:**
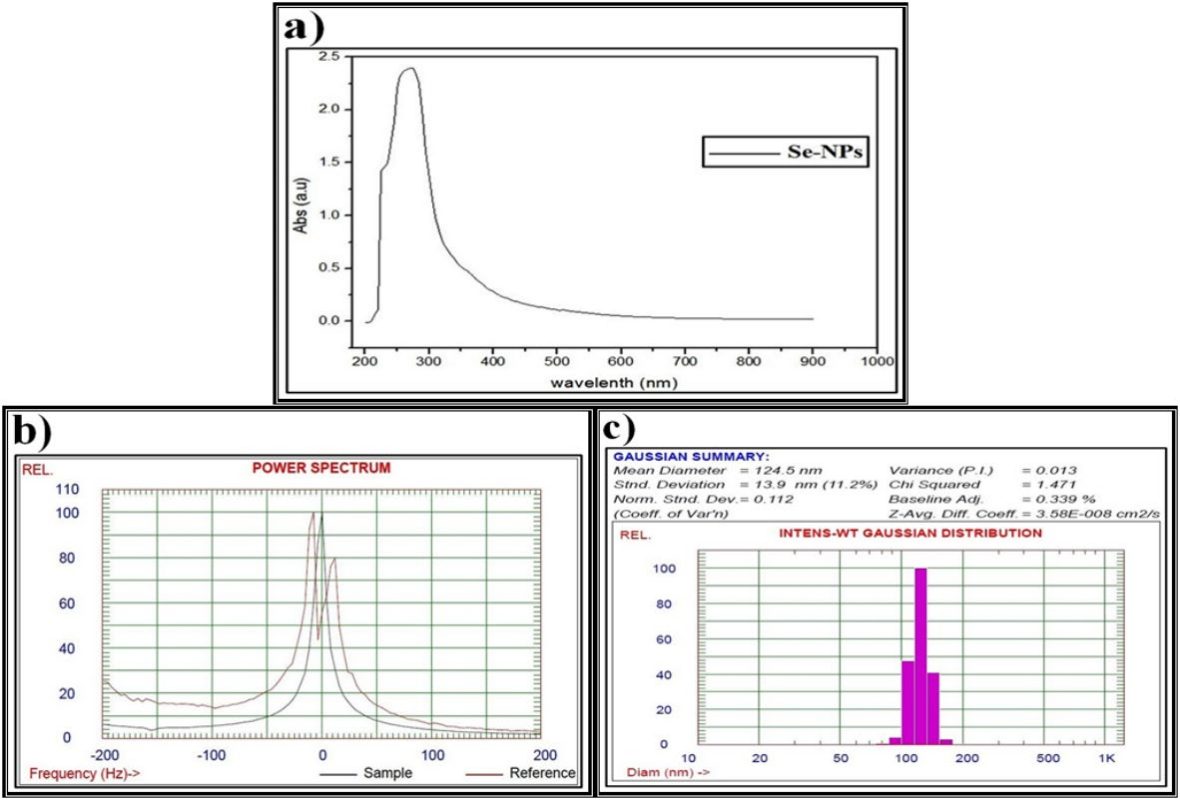
Data of the biosynthesized selenium nanparticles (Se-NPs) using aqueous *S. alba* seeds extract and characterized by (**a**) Ultraviolet-visible (UV-VIS) spectroscopy, (**b**) Zeta potential, (**c**) Dynamic light scattering (DLS).

**Fig. 10:**
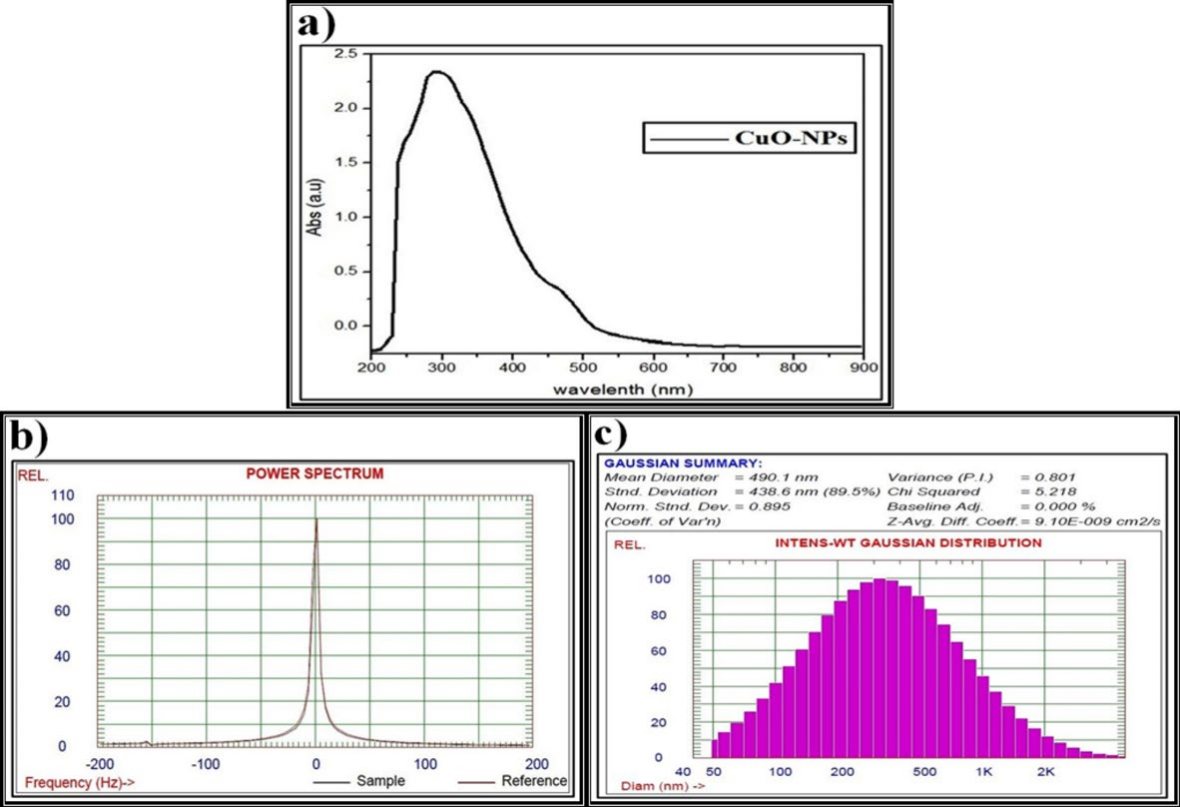
Data of the biosynthesized copper oxide nanoparticles (CuO-NPs) using aqueous *S. alba* seeds extract and characterized by (**a**) Ultraviolet-visible (UV-VIS) spectroscopy, (**b**) Zeta potential, (**c**) Dynamic light scattering (DLS).

As shown in (Fig. 9), the UV spectra, zeta potential, and DLS pattern confirm the formation of Se-NPs biosynthesized using aqueous *S. alba* seeds extract. The data presented in (Fig. 10) confirm the biosynthesis of CuO-NPs using aqueous *S. alba* seeds extract as discussed before.

### The median lethal dose (LD_50_)

**Fig. 11:**
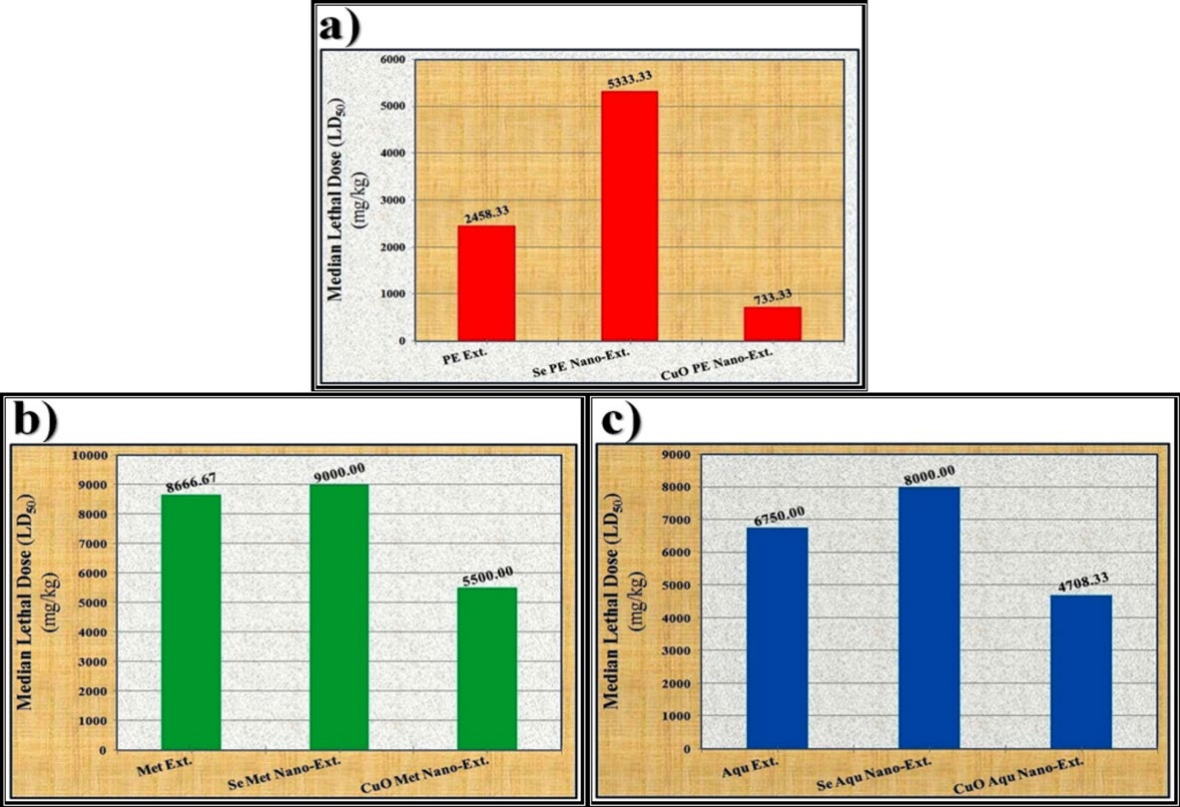
Data of the median lethal doses (LD_50_) of different *S. alba* seeds metal nano-extracts and compared to the native extracts. (**a**) P. Ether extract, (**b**) Methanolic extract and (**c**) Aqueous extract.

It was found that the methanolic *S. alba* seed extract was safer than the other native extracts (P. ether and aqueous) when administered orally to the experimental animals (mice). The data presented in Fig. 11a show that the Se P. ether *S. alba* seed nano-extract appeared safer compared to the native P. ether extract itself. On the contrary, the CuO P. ether *S. alba* seed nano-extract showed higher toxicity compared to the Se P. ether nano-extract and the native P. ether extract itself.

Regarding the methanolic *S. alba* seed extract (Fig. 11b), it was found that the Se methanolic nano-extract appeared safer than the native methanolic extract itself. In contrast, the CuO methanolic nano-extract showed higher toxicity compared to the Se methanolic nano-extract and the native methanolic extract itself. Regarding the aqueous extract (Fig. 11c), it was noticed that the Se aqueous nano-extract appeared safer compared to the native extract itself. In contrast, the CuO nano-extract appeared more toxic compared to the Se nano-extract and the native aqueous extract itself.

The overall results related to the oral administration of the native extracts and metal nano-extracts showed that the safety of all extracts increased when they were utilized for the biosynthesis of Se-NPs, and their toxicity increased when they were utilized for the biosynthesis of CuO-NPs. This is consistent with Abadi *et al*.^114^, who postulated that the presence of the biocompounds with the biosynthesized Se-NPs enhances biological functionality and reduces their toxicity. The safety of the biosynthesized Se-NPs might be related to their higher unique biocompatibility and biodegradability properties than those of their organic and inorganic analogs^115^. Moreover, the activity of the glutathione peroxidase enzyme did not differ in the liver and kidney between Se-methionine and Se-NPs. Therefore, the biosynthesized Se-NPs displayed less immediate liver injury compared to Se-methionine^116^.

Administration of the phyto-synthesized M-NPs was orally safer than the native extracts due to their biodistribution in organs and tissues depending on their sizes and doses, which stimulate their direct interactions in biological systems with biomacromolecules^117^. Additionally, their safety might be related to the renal efficiency in removing them from the body with a lower degradation rate^118^.

## Conclusion

Among the various extracts of *S. salba* seeds, the methanolic extract demonstrates the highest concentrations of total polyphenols, condensed tannins, and total flavonoid compounds, while the aqueous extract follows in concentration, and the petroleum ether extract shows the least. The biosynthesized Se-NPs using methanolic *S. alba* extract showed higher *in vitro* biological and cytotoxic activities compared to those of the biosynthesized CuO-NPs and the corresponding native extract itself. The biosynthesized Se-NPs using petroleum ether, methanolic, and aqueous extracts appeared safer compared to their corresponding native extracts when administered orally. Therefore, the total methanolic extract is a valuable bioresource for biosynthesizing Se-NPs through green nanotechnology, with higher biological efficiency in relation to its metabolite fingerprint.

## Electronic supplementary material

Below is the link to the electronic supplementary material.

Supplementary Material 1

## Data Availability

The manuscript has associated data as supplementary materials.
