# Supplementary material for: Phytochemical analysis and biological study on Sinapis alba L. seeds extract incorporated with metal nanoparticles, in vitro approach
Source: Sci Rep. 2025 Apr 21;15:13782. doi: 10.1038/s41598-025-95347-6 (PMC12012182; doi:10.1038/s41598-025-95347-6)
Supplement: Supplementary file 1 — Supplementary Material 1 [file 41598_2025_95347_MOESM1_ESM.doc]

**Detailed Methodology**

**Spectrophotometric analysis**

***Total polyphenolic compound***

The concentration of total polyphenolic compounds was estimated in the different extracts using the Folin-Ciocalteu reagent with gallic acid as the standard. In this method, 0.1 mL of the extract was diluted to 0.5 mL with distilled water. Then, 0.25 mL of Folin-Ciocalteu reagent was added, followed by 1.25 mL of aqueous sodium carbonate solution. All tubes were vortexed and then incubated for 40 minutes at room temperature. The absorbance of the blue-colored mixtures was recorded at a wavelength of 725 nm against a blank containing 0.5 mL of distilled water instead of the extract. The concentration of total polyphenolic compounds was calculated as a gallic acid equivalent from the calibration curve of various concentrations of gallic acid standard solutions.

***Total condensed tannins***

Concentrations of total condensed tannins were quantified in all prepared extracts. First, it is necessary to extract total tannins by macerating 1gm of plant tissue with acetone (70% v/v): water (tissue weight: vol., 1 : 3), containing ascorbic acid (0.1% w/v). The macerate and washings were centrifuged at 2000 g for 5 min and the liquid supernatant transferred to a separating funnel. The residue was re-extracted (5 x) with acetone (70% v/v). The combined extracts, on saturation with NaCl, separated into a lower aqueous and upper acetone phase. The aqueous phase was re-extracted with the upper (acetone) phase of a NaCl saturated solution of acetone (70% v/v). The combined acetone phases were evaporated to remove acetone. Water (2 mL) was added to the solution and extracted (3 x) with diethyl ether and then (3 x) with ethyl acetate. The aqueous solution (containing condensed tannins) was made up to a standard volume (5 or 10 mL).

The sample volume was 0.5 mL, and the total reaction volume was 5 mL. Vanillin reagent (3 mL) was added to the sample and mixed thoroughly. Concentrated hydrochloric acid (1.5 mL) was added and mixed thoroughly. The reaction mixture was allowed to stand for 15 min at 20 ± 2°C. Absorbance of the samples and blank was determined against water at 500 nm. The concentration of total condensed tannins was determined from the curve plotted between absorbance and a series of different concentrations of purified tannins (standard).

***In vitro* biological activities**

***Antioxidant activity***

The total antioxidant capacity (TAC) was determined by analyzing the green phosphate/Mo^5+^ complex at a wavelength (λ) of 695 nm. Samples were mixed with a reagent solution containing 0.3 N sulfuric acid, 28 mM sodium phosphate, and 4 mM ammonium molybdate. Methanol (80%) was used in place of the sample for the blank. The tubes were sealed and incubated in a boiling water bath for 90 minutes. After cooling to room temperature, the absorbance was measured at 695 nm against the blank. Ascorbic acid was used at the same concentrations as a standard. The antioxidant capacity was expressed as mg gallic acid equivalent per gram weight.

The iron reducing power was determined as µg/mL. In brief, 1mL of the tested sample (at each concentration) was combined with 1mL of 200mM sodium phosphate buffer (pH 6.6) and 1mL of 1% potassium ferricyanide. The mixture was then incubated at 50°C for 20 minutes, followed by the addition of 1mL of trichloroacetic acid (10%). After centrifugation at 2000rpm for 10 minutes, the upper layer solution (2.5 mL) was mixed with 2.5 mL of double deionized water and 1mL of fresh ferric chloride (0.1%). The absorbance was measured at 700nm against a blank prepared without the sample. Ascorbic acid was used at the same concentrations as a standard. A high absorbance at 700nm indicates a higher reducing power in the reaction mixture.

***Scavenging activity***

The 1,1-Diphenyl-2-picryl-hydrazyl (DPPH) radical scavenging activities were evaluated. An antioxidant substance capable of donating a hydrogen atom to a solution containing DPPH- can reduce the stable free radical, causing the solution to change color from violet to pale yellow. The remaining DPPH- radical was quantified by measuring the intensity of a light-purple colored DPPH methanol solution in the visible range at 518 nm using a spectroscopic method. Two milliliters of a DPPH solution (100 µM) in ethanol were mixed with 2 mL of the sample (at each concentration). The reaction mixture for each concentration was thoroughly vortexed and then incubated in the dark at room temperature for 30 minutes. The absorbance was then measured spectrophotometrically at 518 nm against a blank (ethanol). For the control, 2 mL of ethanol was added instead of the sample and run simultaneously with the test. Ascorbic acid was used at the same concentrations as a positive control. Percent inhibition of the DPPH free radical was calculated.

During the procedure used for assaying the 2,2'-azinobis-(3-ethylbenzothiazoline-6-sulfonic acid) (ABTS), Stock solutions included ABTS solution (7 mM) and potassium persulfate solution (2.4 mM). The working solution was prepared by mixing the two stock solutions in equal quantities and allowing them to react at room temperature in a dark place for 14 hours. The solution was then diluted by mixing 1 mL of ABTS solution with 60 mL of methanol to obtain an absorbance of 0.706 ± 0.01 units at 734 nm using a spectrophotometer. Fresh ABTS solution was prepared for each assay. The tested samples (at each concentration) were allowed to react with 1 mL of the ABTS solution, and the absorbance was taken at 734 nm after 7 minutes using a spectrophotometer. The ABTS scavenging capacities of the samples were compared with that of ascorbic acid (at the same concentrations).

In this investigation, Griess Illosvory reagent was generally modified by using napthyl ethylene diamine dihydrochloride (0.1% w/v) instead of 1-napthylamine (5%). The reaction mixture (3 mL) containing 2 mL of 10 mM sodium nitroprusside, 0.5 mL saline phosphate buffer and 0.5 mL of standard solution or aqueous and ethanolic extracts (500–1000 μg/ mL) were incubated at 25°C for 150 minutes. After incubation, 0.5 mL of the reaction mixture was mixed with 1 mL sulfanilic acid reagent (0.33% in 20% glacial acetic acid) and allowed to stand for 5 minutes for the completion of the reaction of diazotization. After this, a further 1 mL of the napthyl ethylene diamine dihydrochloride was added, mixed and was allowed to stand for 30 minutes at 25°C. The concentration of nitrite was assayed at 546 nm and was calculated with the control absorbance of the standard nitrite solution (without extracts or standards, but the same condition should be followed). Here buffer was used as blank solution and Ascorbic acid was taken as standard solution.

***Anti-diabetic activity***

This assay involved calculating the inhibition percentage (%) of α-amylase enzyme using Acarbose as the standard drug. During the assay, 0.5 mL of each sample (at each concentration) was combined with 0.5 mL of α-amylase solution (0.5 mg/mL) and buffer (Na_2_HPO_4_/NaH_2_PO_4_ (0.02 M), NaCl (0.006 M) at pH 6.9). The mixture was then left at room temperature for 10 minutes before adding 200 μL of starch solution (1% in water (w/v) buffer (Na_2_HPO_4_/NaH_2_PO_4_ (0.02 M), NaCl (0.006 M) at pH 6.9)). The reaction was stopped by adding 200 μL of DNSA (coloring) reagent (12 g of sodium potassium tartrate tetrahydrate in 8.0 mL of 2 M NaOH and 20 mL of 96 mM of DNSA solution). The test tubes were then placed in a boiling water bath (100 °C) for 10 minutes and the mixture was cooled to room temperature and diluted with 5 mL of distilled water. The absorbance was measured at 540 nm using a UV-Visible spectrophotometer.

The inhibition percentage (%) of the α-glucosidase enzyme was determined with Acarbose as the standard drug. Five μL of the α-glucosidase solution (10 units mL^−1^, 0.1moll^−1^ potassium phosphate buffer, pH 6.8) was pre-mixed with 10 μL of each sample (at each concentration). After incubation at 37.5 °C for 20 minutes, 10 μL of p-nitro phenyl glucopyranoside (pNPG, 10 mmoll^−1^) as a substrate was added to the mixture to start the reaction. The reaction mixture was then incubated at 37.5°C for 30 minutes, followed by the addition of 650 μL of 1 moll^−1^ Na_2_CO_3_ solution to terminate the reaction. The amount of released product (p-nitro phenol) was measured at 410 nm using a UV spectrometer (UV-2550, Shimadzu, Japan) to estimate the enzymatic activity.

***Anti-acetylcholinesterase activity***

In this study, we assessed the inhibition percentage of the acetylcholinesterase (AChE) enzyme with donepezil as the standard drug. For each run, 5 µL of Acetylthiocholine (ATCh) at a concentration of 0.5 mM, 5 µL of 5,5’-dithiobis-2-nitrobenzoic acid (DTNB) at a concentration of 0.03 mM, and 5 µL of each sample (at each concentration) were added to a flat bottom 96-well plate. The mixture was then incubated for 10 minutes at 30 °C. After incubation, 5 µL of AChE at a concentration of 0.3 U/mL was added to start the reaction, and the absorbance was measured at 412nm. A control run was also performed, which included all the components except for the test sample. The median inhibitory concentration (IC_50_) of each tested sample was calculated by plotting a curve using a series of sample concentrations against the percent of AChE inhibition.

***Anti-arthritic activity***

This assay involved determining the inhibition percentage (%) of protein denaturation and the activity of proteinase enzyme using diclofenac sodium as the standard non-steroidal anti-inflammatory drug. The percentage of protein denaturation inhibition was measured by mixing 0.5mL of the test control solution, prepared by combining 0.45 mL of bovine serum albumin (BSA) (5% w/v aqueous solution) with 0.05 mL of distilled water. Then, 0.05 mL of each sample (at each concentration) was added to 0.45 mL of distilled water to form the product control (0.5 mL). The pH value in all prepared solutions was adjusted to 6.3 using HCl (1N). All the samples were incubated at 37 ^o^C for 20 min, and the temperature was then increased to 57 ^o^C, maintaining the samples at that degree for 3 min. After cooling, 2.5 mL of phosphate buffer was added to the prepared solutions. The absorbance was determined at 416 nm using a UV-Visible spectrophotometer. The percentage of protein denaturation inhibition can be calculated.

The inhibition percentage of proteinase enzyme was assessed by combining 1 mL of each sample (at each concentration) with a reaction mixture containing 0.06 mg trypsin dissolved in 1 mL of 20 mM Tris HCl buffer (pH 7.4). The mixture was then incubated for 5 minutes at 37°C, followed by the addition of 1 mL of casein (0.8% w/v). After an additional 20 minutes of incubation, 2 mL of perchloric acid (70%) was added to stop the reaction. The cloudy suspension was then centrifuged, and the absorbance of the supernatant was measured at 210 nm against buffer as the blank. The IC_50_ of each tested sample was calculated by plotting a curve using a series of sample concentrations against the percent of proteinase inhibition.

***The anti-inflammatory activity***

*In vitro* anti-inflammatory activities were evaluated through the inhibition of two isoenzymes cyclooxygenase-1 (COX-1) and cyclooxygenase-2 (COX-2) (ovine/human), along with the 5-LOX enzyme (human recombinant). The inhibition percentages of COX-1 and COX-2 were measured using the COX-1 and COX-2 kit. The inhibition percentages of 5-Lipoxygenase (5-LOX) were assessed using the 5-LOX kit. The IC_50_ was determined *via* linear regression.

***Cytotoxic activity***

It was assayed against human hepatocellular (HEPG-2), colon carcinoma (CACO-2), and lung cancer (A549) cells and compared to normal human fibroblast (BJ-1) cell line using 3-(4,5-dimethythiazol-2-yl)-2,5-diphenyl tetrazolium bromide (MTT) assay by determining the optical density (OD) at 570 nm. The cells were dispensed in a 96-well sterile microplate (3 × 10^4^ cells/ well), followed by their incubation at 37 °C with a series of different concentrations of 10 μL of each compound or doxorubicin (positive control, in DMSO) for 48 h in a serum free medium prior to the MTT assay. Subsequently, the media were carefully removed, and 40 μL of MTT (2.5 mg/mL) was added to each well and then incubated for an additional 4 h. Purple formazan dye crystals were solubilized by the addition of 200 μL of DMSO. The absorbance was measured at 570 nm using a SpectraMax Paradigm Multi-Mode microplate reader. The relative cell viability was expressed as the mean percentage of viable cells relative to the untreated control cells. All experiments were conducted in triplicate. Percent of the cell-growth inhibition (%) and the IC_50_ was calculated using IC_50_ calculation software.

***The enzymatic activity***

Activities of caspase-3 were measured by enzyme-linked immunosorbent assay (ELISA) using the Invitrogen caspase-3 (Active) (human) ELISA kit (96 tests) from Invitrogen Corporation, following the manufacturer's instructions. Activities of Bcl-2 were measured using the Invitrogen Zymed Bcl-2 ELISA Kit (96 tests) from Invitrogen Corporation, following the manufacturer's instructions.

**~~Supplementary Table 1~~** ~~Percentages, physical and chemical characters of the P. ether, methanolic and aqueous~~ *~~S. alba~~* ~~seeds extract utilized for biosynthesis of Se- and CuO-NPs.~~

| **Character** | **P. ether** | | | **Methanolic** | | | **Aqueous** | | |
| --- | --- | --- | --- | --- | --- | --- | --- | --- | --- |
|  | **Ext.** | | **Nano-Ext.** | **Ext.** | **Nano-Ext.** | | **Ext.** | **Nano-Ext.** | |
| Percentage | 46% | | | 28% | | | 19% | | |
| **Physical characters** | | | | | | | | | |
| Color | Yellow | | | Dark yellow | | | Dark yellow | | |
| Condition | Oily | | | Gummy | | | Gummy | | |
| **Constituents** | | | | | | | | | |
| Carbohydrate &/or glycosides | - | − | | + | | + | + | | + |
| Flavonoids | - | − | | + | | ++ | + | | + |
| Saponins | - | − | | - | | − | - | | − |
| Tannins | - | − | | + | | + | + | | − |
| Sterols &/or terpenes | ++ | + | | - | | − | - | | − |
| Alkaloids &/or nitrogenous compounds | - | − | | - | | − | + | | + |
| Coumarins | - | − | | - | | − | - | | − |
| Anthraquinones | - | − | | - | | − | - | | − |

(-): Absent, (+): Present, (++): Appreciably present.

**Supplementary Table 2a** GC/MS analysis of the lipoidal constituents in petroleum ether *S. alba* seeds extract.

| **Class** | **Compound** | **Mol. formula** | **Mol. weight** | **BP** | **Percentage** | **Total percentage** |
| --- | --- | --- | --- | --- | --- | --- |
| **Saturated hydrocarbons** | Isopropylcyclopentane | C_8_H_16_ | 112 | 68 | **0.84** | **21.22** |
|  | n-Octane | C_8_H_18_ | 114 | 43 | **1.06** |  |
|  | Cycloheptylcycloheptane | C_14_H_26_ | 194 | 55 | **1.32** |  |
|  | n-Decylcyclopentane | C_15_H_30_ | 210 | 41 | **0.85** |  |
|  | n-Decane | C_10_H_22_ | 142 | 43 | **3.95** |  |
|  | 2,5,6-Trimethyldecane | C_13_H_28_ | 184 | 57 | **1.03** |  |
|  | n-Dodecane | C_12_H_26_ | 170 | 43 | **4.67** |  |
|  | n-Docosane | C_22_H_46_ | 310 | 43 | **3.21** |  |
|  | 5-Propyltridecane | C_16_H_34_ | 226 | 57 | **1.63** |  |
|  | 2,6,11-Trimethyldodecane | C_15_H_32_ | 212 | 57 | **0.69** |  |
|  | 2,6,10-Trimethyltetradecane | C_17_H_36_ | 240 | 57 | **1.97** |  |
| **Unsaturated hydrocarbons** | 1-Decyne | C_10_H_18_ | 138 | 41 | **3.26** | **15.39** |
|  | n-Hexadecane | C_16_H_34_ | 226 | 41 | **1.09** |  |
|  | Heptadec-8-ene | C_17_H_34_ | 238 | 55 | **1.68** |  |
|  | 1-Nonadecene | C_19_H_38_ | 266 | 55 | **3.64** |  |
|  | 5-Eicosene | C_20_H_40_ | 280 | 55 | **5.72** |  |
| **Fatty alcohols** | 1-Tridecanol | C_13_H_28_O | 200 | 43 | **2.03** | **4.51** |
|  | 14-Methyl-2,15-octadecadien-1-ol | C_19_H_36_O | 280 | 55 | **1.01** |  |
|  | 1,2-Nonadecanediol | C_19_H_40_O_2_ | 300 | 56 | **1.47** |  |
| **Aldehydes** | n-Hexanal | C_6_H_12_O | 100 | 44 | **2.03** | **6.37** |
|  | 2-Heptenal | C_7_H_12_O | 112 | 41 | **3.45** |  |
|  | 16-Octadecenal | C_18_H_34_O | 266 | 82 | **0.89** |  |

**Supplementary Table 2b** GC/MS analysis of the lipoidal constituents in petroleum ether *S. alba* seeds extract.

| **Class** | **Compound** | **Mol. formula** | **Mol. weight** | **BP** | **Percentage** | **Total percentage** |
| --- | --- | --- | --- | --- | --- | --- |
| **Fatty acids** | 9-Tetradecenoicacid | C_14_H_26_O_2_ | 226 | 55 | **1.35** | **15.91** |
|  | n-Pentadecanoic acid | C_15_H_30_O_2_ | 242 | 43 | **1.07** |  |
|  | Linoleic acid | C_18_H_32_O_2_ | 280 | 67 | **4.42** |  |
|  | Oleic Acid | C_18_H_34_O_2_ | 282 | 41 | **3.32** |  |
|  | 9-Hexadecenoicacid | C_16_H_30_O_2_ | 254 | 55 | **0.95** |  |
|  | Palmitic acid | C_16_H_32_O_2_ | 256 | 43 | **3.38** |  |
|  | 9-Tetradecenoicacid | C_14_H_26_O_2_ | 226 | 55 | **1.42** |  |
| **Esters** | 3,7-Dimethyl-2-octenyl  2-methylpropanoate | C_14_H_26_O_2_ | 226 | 57 | **1.03** | **23.94** |
|  | Methyl palmitate | C_17_H_34_O_2_ | 270 | 74 | **4.17** |  |
|  | Ethyl palmitate | C_18_H_36_O_2_ | 284 | 88 | **2.15** |  |
|  | Methyl linoleate | C_19_H_34_O_2_ | 294 | 67 | **4.43** |  |
|  | Methyl oleate | C_19_H_36_O_2_ | 296 | 55 | **3.09** |  |
|  | Methyl stearate | C_19_H_38_O_2_ | 298 | 74 | **2.03** |  |
|  | Ethyl linoleate | C_20_H_36_O_2_ | 308 | 67 | **1.42** |  |
|  | 9-Octadecenoic acid, ethyl ester | C_20_H_38_O_2_ | 310 | 55 | **1.32** |  |
|  | Methyl -9,10-epoxystearate | C_19_H_36_O_3_ | 312 | 55 | **0.71** |  |
|  | Hexadecanoic acid, 2,3dihydroxypropyl  ester | C_19_H_38_O_4_ | 330 | 55 | **0.48** |  |
|  | Octadecanoic acid, 2,3dihydroxypropyl ester | C_21_H_42_O_4_ | 358 | 43 | **0.65** |  |
|  | N-Docosyl n-heptanoate | C_29_H_58_O_2_ | 438 | 131 | **0.83** |  |
|  | Oleic acid, octadecyl ester | C_36_H_70_O_2_ | 534 | 57 | **1.63** |  |
| **Monoterpenes** | Mentha-6,8-diene | C_10_H_16_ | 136 | 93 | **0.29** | **1.71** |
|  | Limonene | C_10_H_16_ | 136 | 68 | **0.64** |  |
|  | Terpinylacetate | C_12_H_20_O_2_ | 196 | 68 | **0.78** |  |

**Supplementary Table 3a** LC-ESI-MS/MS analysis of methanolic *S. salba* seeds extract utilized for biosynthesis of Se- and CuO-NPs.

| **Class** | **Tentative Identification** | **Rt.** | **Mol. ion [M-H]-** | **Molecular formula** | **MS/MS fragments (*m/z*)** | **Native extract** | **Se**  **plant nano-extract** | **CuO**  **plant nano-extract** | **References** |
| --- | --- | --- | --- | --- | --- | --- | --- | --- | --- |
| **Phenolic acid** | Protocatechuic acid | 1.06 | 153 | C_7_H_6_O_4_ | 109[M-H-COO^-^], 94 | + | + | + |  |
|  | Gallic acid | 2.09 | 169 | C_7_H_6_O_5_ | 169, 125[M-H- COO^-^], 107[M-H-COO-H_2_O], 79 | + | + | + | **Sadowska et al. (2023)** |
|  | Caffeic acid | 3.57 | 179 | C_9_H_8_O_4_ | 161[M-H-H_2_O],135[M-H-H_2_O-CO] | + | + | + |  |
|  | Chlorogenic acid | 5.19 | 353 | C_16_H_18_O_9_ | 191 [M-H-Caffeoyl], 179[caffeic acid],161, 135 | + | + | + |  |
|  | Sinapinic acid | 6.29 | 223 | C_11_H_12_O_5_ | 207, 193, 164, 149, 121 | + | + | + | **Jiang et al. (2013)** |
|  | Quinic acid | 7.20 | 191 | C_7_H_12_O_6_ | 191, 173 [M-H-H_2_O], 127 [M-H-2H_2_O-CO], 111, 93, 87, 8 | + | - | - |  |
|  | Ferulic acid | 10.41 | 193 | C_10_H_10_O_4_ | 149 [M-H-CO2],178 [M-H-CH_3_],134 [M-H-CO_2_-CH_3_] | + | + | + | **Rasera et al. (2019)** |
| **Flavone** | Luteolin | 21.01 | 285 | C_15_H_10_O_6_ | 257 [M-H-CO], 241[M-H-COO^-^ ], 217 [M-H-C_3_O_2_], 199 [M-H-C_2_H_2_O-COO^-^], 151 [1,3 A], 133 [1,3 B] | + | + | + | **Sadowska et al. (2023)** |
| [**Flavanone**](https://en.wikipedia.org/wiki/Flavanone) | Naringenin | 9.13 | 271 | C_15_H_12_O_5_ | 253 [M-H-H_2_O], 227 [M-H-COO^-^], 177, 151, 119 | + | + | + | **Martinović et al. (2020)** |
| [**Flavanol**](https://en.wikipedia.org/wiki/Flavan-3-ol) | Gallocatechin | 12.98 | 305 | C_15_H_14_O_7_ | 287 [M-H-H_2_O], 261 [M-H-C_2_H_4_O], 219 [C_12_H_11_O_4_], 179 [M-H-126]ring B fission, 167, 137, 125 [ring B] | + | - | - |  |

**Supplementary Table 3b** LC-ESI-MS/MS analysis of methanolic *S. salba* seeds extract utilized for biosynthesis of Se- and CuO-NPs.

| **Class** | **Tentative Identification** | **Rt.** | **Mol. ion [M-H]-** | **Molecular formula** | **MS/MS fragments (*m/z*)** | **Native extract** | **Se**  **plant nano-extract** | **CuO**  **plant nano-extract** | **References** |
| --- | --- | --- | --- | --- | --- | --- | --- | --- | --- |
|  | Epicatechin | 23.27 | 289 | C_15_H_14_O_6_ | 245 [M-H- C_2_H_4_O], 205, 245, 221, 203, 151, 109 | + | + | + | **Jiang et al. (2013)** |
| **Flavonol** | Limocitrin (4',5,7- trihydroxy-3',8- dimethoxyflavonol) | 7.64 | 345 | C_17_H_14_O_8_ | 330 [M-H-CH_3_],315 [M-H-2CH_3_],276 [M-H-3H_2_O-CH_3_] | + | - | - |  |
|  | Kaempferol | 9.01 | 285 | C_15_H_10_O_6_ | 267[M-H-H_2_O],257[M-H-CO], 227 [M-H-2CHO] | + | + | + | **Jiang et al. (2013)**  **& Martinović et al. (2020)** |
|  | Quercetin | 10.83 | 301 | C_15_H_10_O_7_ | 283[M-H-H_2_O],255[M-H-H_2_O-CO], 179, 151 | + | + | + |  |
| **Methylated flavonol** | Isorhamntin | 10.98 | 315 | C_16_H_12_O_7_ | 301 [M-H-CH_3_],  297[M-H-H_2_O],269[M-H-H_2_O-CO] | + | + | + |  |
| **Methylated flavone** | Sinensetin | 20.34 | 371 | C_20_H_20_O_7_ | 356 [M-H-CH_3_], 355 [M-H-CH_4_], 341[M-H-2CH_3_], 337 [M-H-CH_4_- H_2_O], 310 [M-H-CH_3_-CO-H_2_O) | + | + | + |  |
| **Flavonoid** [**glycoside**](https://www.bing.com/ck/a?!&&p=0b791873ead7102eJmltdHM9MTY5MzA5NDQwMCZpZ3VpZD0xYjhjYTRiYy1hNTc2LTYxNDktMTkyMi1iNTVlYTQwYTYwYTQmaW5zaWQ9NTQ5NA&ptn=3&hsh=3&fclid=1b8ca4bc-a576-6149-1922-b55ea40a60a4&u=a1L3NlYXJjaD9xPUdseWNvc2lkZSUyMHdpa2lwZWRpYSZmb3JtPVdJS0lSRQ&ntb=1)**s** | Apigenin 7-*O*- glucoside | 10.97 | 431 | C_21_H_20_O_10_ | 413 [M-H-H_2_O],269 [M-H-Glu] | + | + | + | **Sadowska et al. (2023)** |
|  | Kaempferol 3- *O*-glucoside | 15.62 | 447 | C_21_H_20_O_11_ | 285[M-H-Glu],255[M-H-Glu- CH_2_O],227[M-H- Glu- 2CHO] | + | + | + |  |
|  | Isoquercetin (quercetin 3- *O*-glucoside) | 17.12 | 463 | C_21_H_20_O_12_ | 301[M-H-Glu],271[M-H-Glu-CH_3_-CO_2_H],  255, 178, 151 | + | + | - |  |

**Supplementary Table 3c** LC-ESI-MS/MS analysis of methanolic *S. salba* seeds extract utilized for biosynthesis of Se- and CuO-NPs.

| **Class** | **Tentative Identification** | **Rt.** | **Mol. ion [M-H]-** | **Molecular formula** | **MS/MS fragments (*m/z*)** | **Native extract** | **Se**  **plant nano-extract** | **CuO**  **plant nano-extract** | **References** |
| --- | --- | --- | --- | --- | --- | --- | --- | --- | --- |
|  | Isorhamnetin3-*O*-rutinoside | 17.39 | 623 | C_28_H_32_O_16_ | 477[M-146], 315[M-H-Rut], 271, 255, 243 | + | + | + |  |
|  | quercetin 3,7-*O*-dirhamnoside | 18.95 | 593 | C_27_H_30_O_15_ | 447[M-H-Rha],301[M-H-2Rha],  283[M-H-2Rha - H_2_O] | + | + | + |  |
|  | Rutin (quercetin3-*O*-rutinoside) | 21.70 | 609 | C_27_H_30_O_16_ | 301[M-H-Rut],271[M-H- Rut -CH_2_O],255[M-H- Rut-CO-H_2_O],179,151 | + | + | + | **Jiang et al. (2013)**  **& Martinović et al. (2020)** |
|  | Myricetin3-O-rhamnoside | 21.89 | 463 | C_21_H_20_O_12_ | 445[M-H-H_2_O],  317[M-H-Rha],299 [M-H-Rha -H_2_O] | + | + | + |  |
|  | Myricetin7-*O*- glucoside | 22.07 | 479 | C_21_H_20_O_13_ | 461[M-H-H_2_O],317[M-H-Glu],299 [M-H-Glu-H_2_O] | + | + | + |  |

**Supplementary Table 4a** LC-ESI-MS/MS analysis of aqueous *S. salba* seeds extract utilized for biosynthesis of Se- and CuO-NPs.

| **Class** | **Tentative Identification** | **Rt** | **Mol. ion [M-H]-** | **Molecular formula** | **MS/MS fragments** | **Native extract** | **Se**  **plant nano-extract** | **CuO**  **plant nano-extract** | **References** |
| --- | --- | --- | --- | --- | --- | --- | --- | --- | --- |
| **Phenolic acid derivatives** | Ferulic acid (hydroxycinnamic acid) | 1.48 | 193 | C_10_H_10_O_4_ | 178 [M-H-CH_3_], 161 [M-H-OCH_3_], 149 [M-H-COO^-^], 133 | + | + | + | **Rasera et al. (2019)** |
|  | 3,4-Dimethoxy cinnamic acid | 1.93 | 207 | C_11_H_12_O_4_ | 193, 178[M-H-C_2_H_5_], 164, 150 | + | + | + |  |
|  | Ellagic acid | 3.40 | 301 | C_14_H_6_O_8_ | 283[M-H-H_2_O], 257 [M-H-COOH], 185[M-H- 2CO_2_−CO] | + | + | + |  |
|  | Methyl gallate (Gallicin) | 3.49 | 183 | C_8_H_8_O_5_ | 168[M-H-CH_3_], 140, 124 [M-H-COOCH_3_],111 | + | + | + |  |
|  | Hydroxymethoxy benzoic acid | 5.73 | 167 | C_8_H_8_O_4_ | 152 [M-H- CH_3_], 123 [M-H-COO^-^], 108 [M-H-CH_3_- COO^-^] | + | - | - |  |
|  | Caffeoylhexoside | 5.82 | 341 | C_15_H_18_O_9_ | 179[M-H-caffoeyl],161[M-H- caffoeyl-H_2_O],135[M-H-caffoeyl-CO_2_] | + | + | - |  |
|  | Hydroxydimethoxy benzoic acid | 6.11 | 197 | C_9_H_10_O_5_ | 182 [M-H- CH_3_], 153 [M-H- COO^-^], 137, 125, 113, 85 | + | + | + |  |
|  | *P*-Coumaroylquinic acid | 7.16 | 337 | C_16_H_18_O_8_ | 191[M-H-coumaroyl], 163[coumaric acid] | + | - | + |  |
| **Flavonoid** [**glycoside**](https://www.bing.com/ck/a?!&&p=0b791873ead7102eJmltdHM9MTY5MzA5NDQwMCZpZ3VpZD0xYjhjYTRiYy1hNTc2LTYxNDktMTkyMi1iNTVlYTQwYTYwYTQmaW5zaWQ9NTQ5NA&ptn=3&hsh=3&fclid=1b8ca4bc-a576-6149-1922-b55ea40a60a4&u=a1L3NlYXJjaD9xPUdseWNvc2lkZSUyMHdpa2lwZWRpYSZmb3JtPVdJS0lSRQ&ntb=1)**s** | Apigenin 7-*O*-glucoside | 5.48 | 431 | C_21_H_20_O_10_ | 413 [M-H-H_2_O],269 [M-H-Glu] | + | + | + | **Sadowska et al. (2023)** |
|  | Kaempferol-3- *O*-diglucoside | 7.81 | 609 | C_27_H_30_O_16_ | 429,319, 285[M-H-diglucoside], 255[M-H- diglucoside -CH_2_O],227[M-H-diglucoside- 2CHO] | + | + | + | **Jiang et al. (2013)** |

**Supplementary Table 4b** LC-ESI-MS/MS analysis of aqueous *S. salba* seeds extract utilized for biosynthesis of Se- and CuO-NPs.

| **Class** | **Tentative Identification** | **Rt** | **Mol. ion [M-H]-** | **Molecular formula** | **MS/MS fragments** | **Native extract** | **Se**  **plant nano-extract** | **CuO**  **plant nano-extract** | **References** |
| --- | --- | --- | --- | --- | --- | --- | --- | --- | --- |
|  | Kaempferol 3- *O*-xyloside | 7.91 | 417 | C_20_H_18_O_10_ | 285[M-H-xyloside], 255 [M-H-xyloside-CH_2_O], 227[M-H- xyloside-2CHO] | + | + | + |  |
|  | Kaempferol3- *O*-rutinoside | 11.01 | 593 | C_27_H_30_O_15_ | 285[M-H-rutinoside], 255[M-H-rutinoside-CH_2_O],227[M-H-rutinoside-2CHO] | + | + | + |  |
|  | Vitexin-2''-*O*-rhamnoside | 13.64 | 577 | C_27_H_30_O_14_ | 413, 311, 293, 269[M-H-rutinoside] | + | - | + |  |
|  | Quercetin 3- *O*-arabinoside | 15.26 | 433 | C_20_H_18_O_11_ | 301[M-H-arabinose], 269, 256[M-H-arabinose-CO_2_H] | + | + | + |  |
|  | Luteolin-8-C-β-D-glucopyranoside7-*O*-rhamnosid | 22.44 | 593 | C_27_H_30_O_15_ | 473[M-H-120],327[M-H-120-146],298 | + | - | - |  |
| **Aliphatic glucosinolates** | Glucobrassicanapin (4-Pentenyl glucosinolate) | 1.56 | 386 | C_12_H_21_NO_9_S_2_ | 307, 146, 136 | + | + | + | **Lietzow (2021)** |
|  | Gluconapin (butenylglucosinolate) | 8.95 | 372 | C_11_H_19_NO_9_S_2_ | 249, 136, 97, 81, 65 | + | + | + |  |
|  | Progoitrin (2-Hydroxy-3-butenyl-glucosinolate) | 12.40 | 388 | C_11_H_19_NO_10_S_2_ | 259, 195, 136, 97, 75 | + | + | + | **Velíšek et al. (1995)** |
|  | Sinigrin (allylglucosinolate) | 12.78 | 358 | C_10_H_17_NO_9_S_2_ | 169, 147, 129, 97, 75 | + | + | + | **Popova and Morra (2014)** |

**Supplementary Table 4c** LC-ESI-MS/MS analysis of aqueous *S. salba* seeds extract utilized for biosynthesis of Se- and CuO-NPs.

| **Class** | **Tentative Identification** | **Rt** | **Mol. ion [M-H]-** | **Molecular formula** | **MS/MS fragments** | **Native extract** | **Se**  **plant nano-extract** | **CuO**  **plant nano-extract** | **References** |
| --- | --- | --- | --- | --- | --- | --- | --- | --- | --- |
| **Aromatic aryl glucosinolates** | Glucotropaeolin (benzyl glucosinolate) | 9.89 | 408 | C_14_H_19_NO_9_S_2_ | 368, 315, 159, 97, 75 | + | + | + | **Lietzow (2021)** |
|  | Gluconasturtiin (phenethylglucosinolate) | 19.01 | 422 | C_15_H_21_NO_9_S_2_ | 298, 135, 97, 65 | + | + | + |  |
|  | Glucosinalbin (4-Hydroxybenzyl glucosinolate) | 20.36 | 424 | C_14_H_19_NO_10_S_2_ | 300, 135, 97, 75 | + | + | + | **Lietzow (2021)** from *B. juncea* |
| **Three Sulfur containing glucosinolates** | Glucoerucin (4-methylthiobutyl glucosinolate) | 7.91 | 420 | C_12_H_23_NO_9_S_3_ | 275, 259, 178, 97, 75 | + | + | + | Identified by **Velíšek et al. (1995)** from *B. juncea* and by **Lietzow (2021)** from *S. alba* |
|  | Glucoibervirin | 10.77 | 406 | C_11_H_21_NO_9_S_3_ | 326, 275, 259, 228, 145 | + | + | + | **Velíšek et al. (1995)** |
|  | Glucoiberin | 18.65 | 422 | C_11_H_21_NO_10_S_3_ | 407, 358, 259, 180, 195, 75 | + | + | + | **Lietzow (2021)** |

**
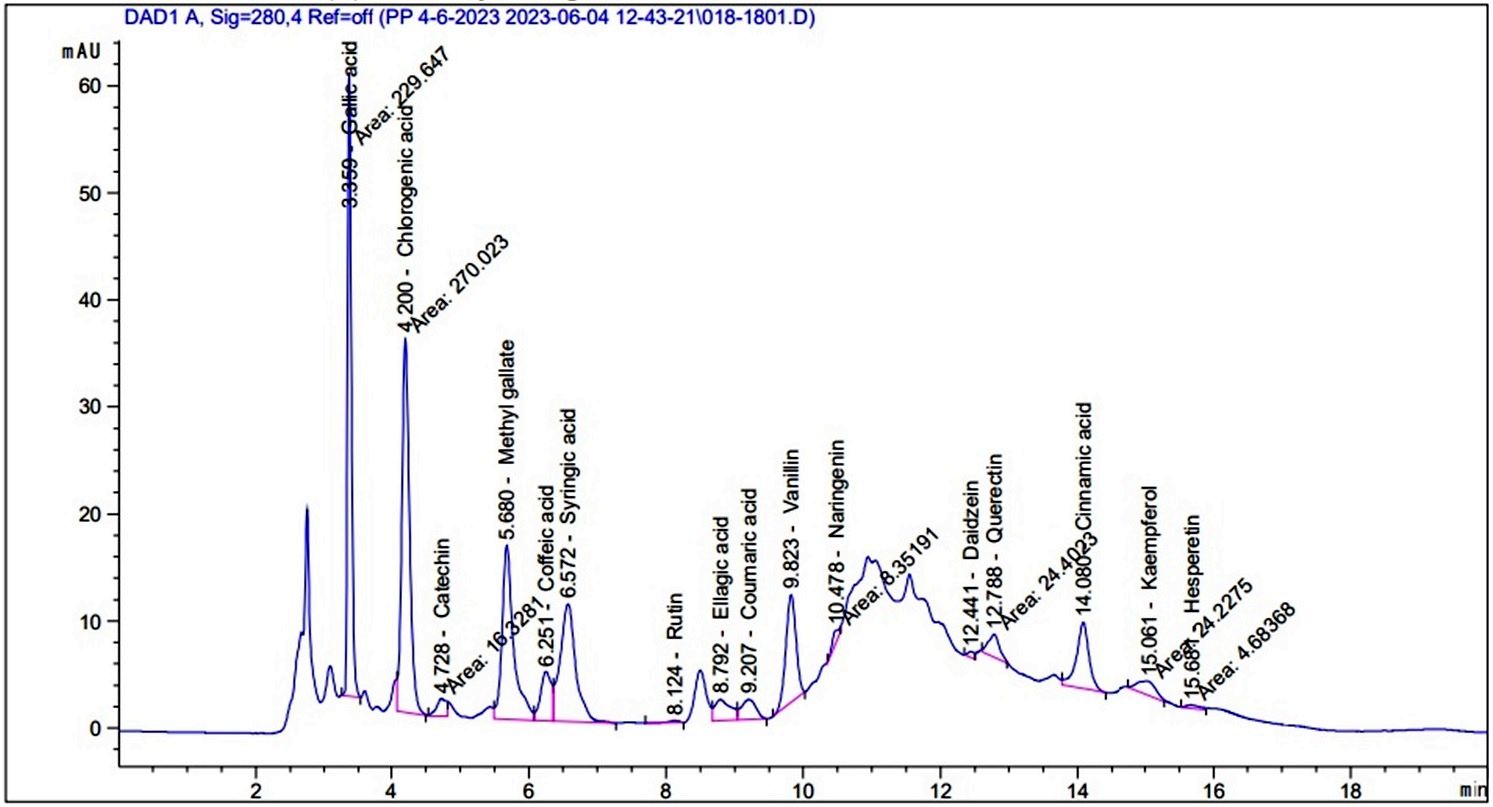
**

**Supplementary Fig. 1** HPLC chromatogram of methanolic *S. alba* seeds extract.

**
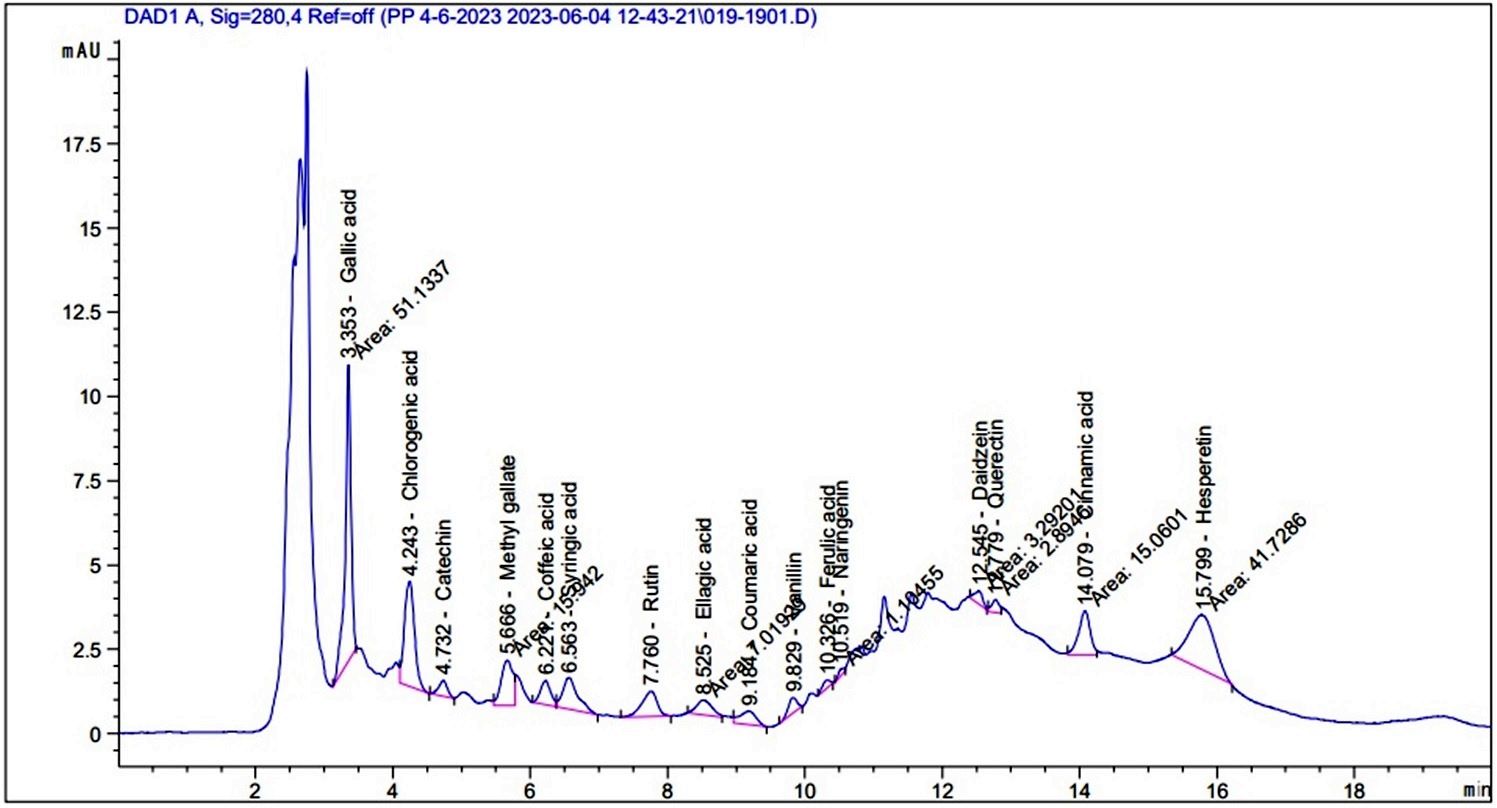
**

**Supplementary Fig. 2** HPLC chromatogram of aqueous *S. alba* seeds extract.

**
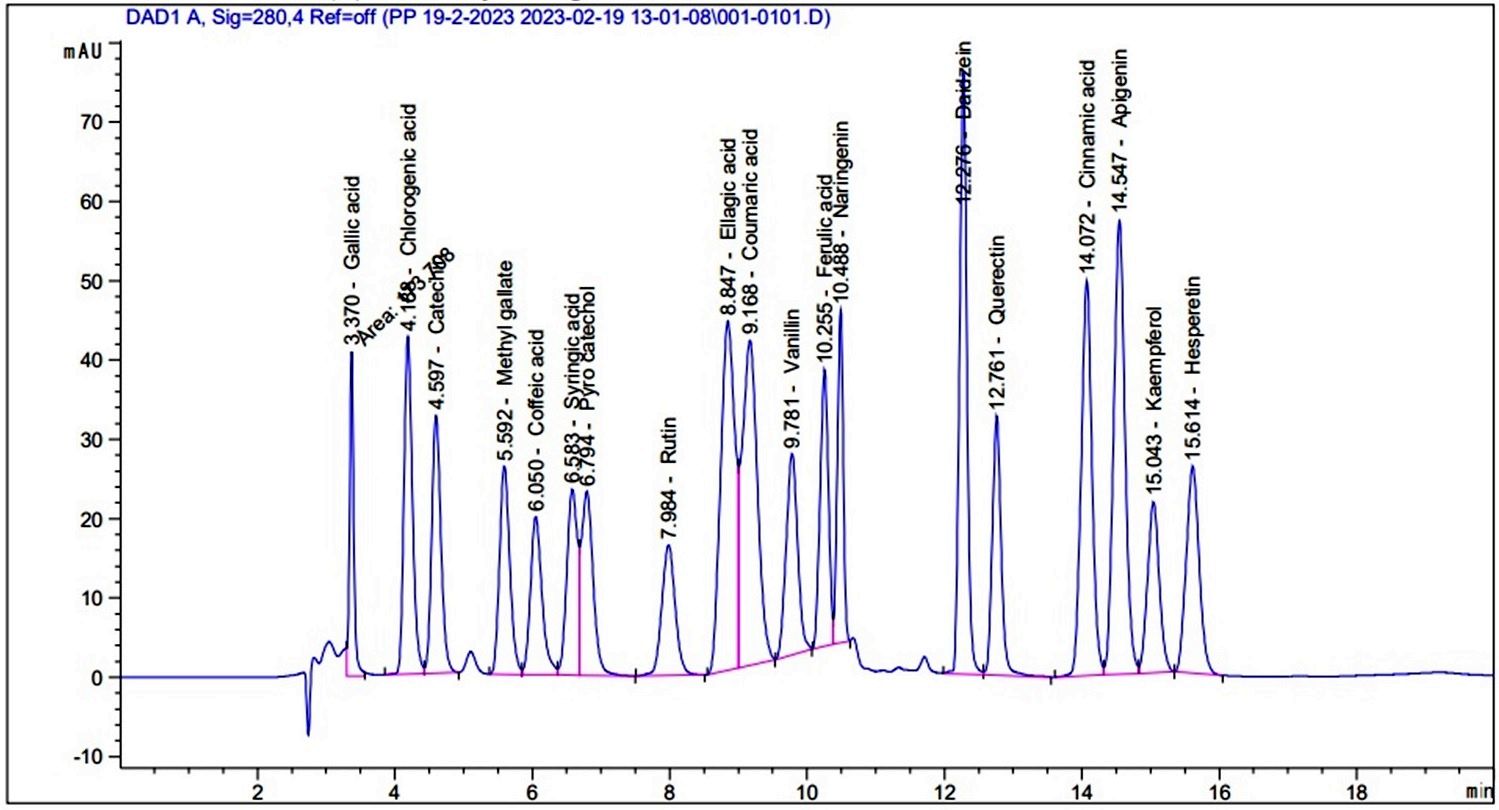
**

**Supplementary Fig. 3** HPLC chromatogram of standard phenolics and flavonoids.
